# Supplementary material for: Endophilin-A3 and Galectin-8 control the clathrin-independent endocytosis of CD166
Source: Nat Commun. 2020 Mar 19;11:1457. doi: 10.1038/s41467-020-15303-y (PMC7081352; doi:10.1038/s41467-020-15303-y)
Supplement: Supplementary file 1 — Supplementary Information [file 41467_2020_15303_MOESM1_ESM.pdf]

Supplementary Information

Endophilin-A3 and Galectin-8 control the clathrin-independent endocytosis of CD166

Renard, Tyckaert *et al.*

Supplementary Figures

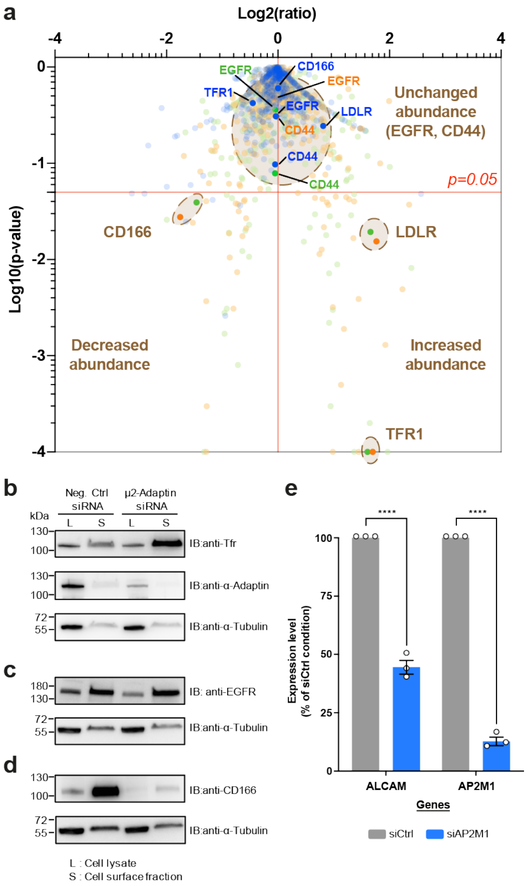

Supplementary Figure 1: Quantitative proteomic analysis of cell surface proteins in conditions of inhibition of clathrin-mediated endocytosis (CME). HeLa cells were treated with control or µ2-adaptin siRNAs. **a,** Mass spectrometry analysis (iTraq) of cell surface proteins. 489 proteins (with Unused score ≥ 2) were identified. Data are presented as a Volcano plot. Two independent analyses, each with two technical replicates of control and µ2-adaptin siRNA conditions. Color code: blue, comparison between technical replicates of control siRNA condition; orange and green, technical replicates of µ2-adaptin siRNA condition compared to the first technical replicate of control condition. Note the strong accumulation (~3.0 to ~3.5-fold) of transferrin receptor (TfR) and LDL receptor (LDLR) at cell surface when clathrin-mediated endocytosis is inhibited, while CD166/ALCAM is strongly downregulated (~2.8 to ~3.4-fold). **b-d,** Western blot analysis of total cell lysates (lane L) and cell surface fractions (lane S) submitted to the previous mass spectrometry analyses (**a**). Anti-α-adaptin antibody was used to show the efficiency of AP-2 complex depletion. α-tubulin was used as a loading control. **b,** Immunoblot with anti-TfR antibody. Note the strong accumulation of TfR (~3- to 4-fold) in the cell surface fraction when CME is inhibited. **c,** Immunoblot with anti-EGFR antibody. Note that CME inhibition does not affect EGFR level in the cell surface fraction. **d,** Immunoblot with anti-CD166 antibody. Note the strong depletion of CD166 (~ 8-fold) in the cell surface fraction. **e,** Quantification of gene expression by qPCR. For each gene – ALCAM/CD166 and AP2M1 – expression level in the control siRNA condition was set to 100%. ACTB (coding for β-actin) was used as a housekeeping gene. Three independent experiments, each with three technical replicates. ****$P < 0.0001$ (Two-tailed unpaired T test with equal variances). Note the 2-fold decrease of ALCAM gene expression upon µ2-adaptin silencing. This decrease of ALCAM gene expression cannot fully explain the strong downregulation of the protein at cell surface previously observed in mass spectrometry (**a**) or Western blot (**d**). Data are mean ± 95% c.i. Source data are provided as a Source Data file and in Supplementary Figure 11 (blots).

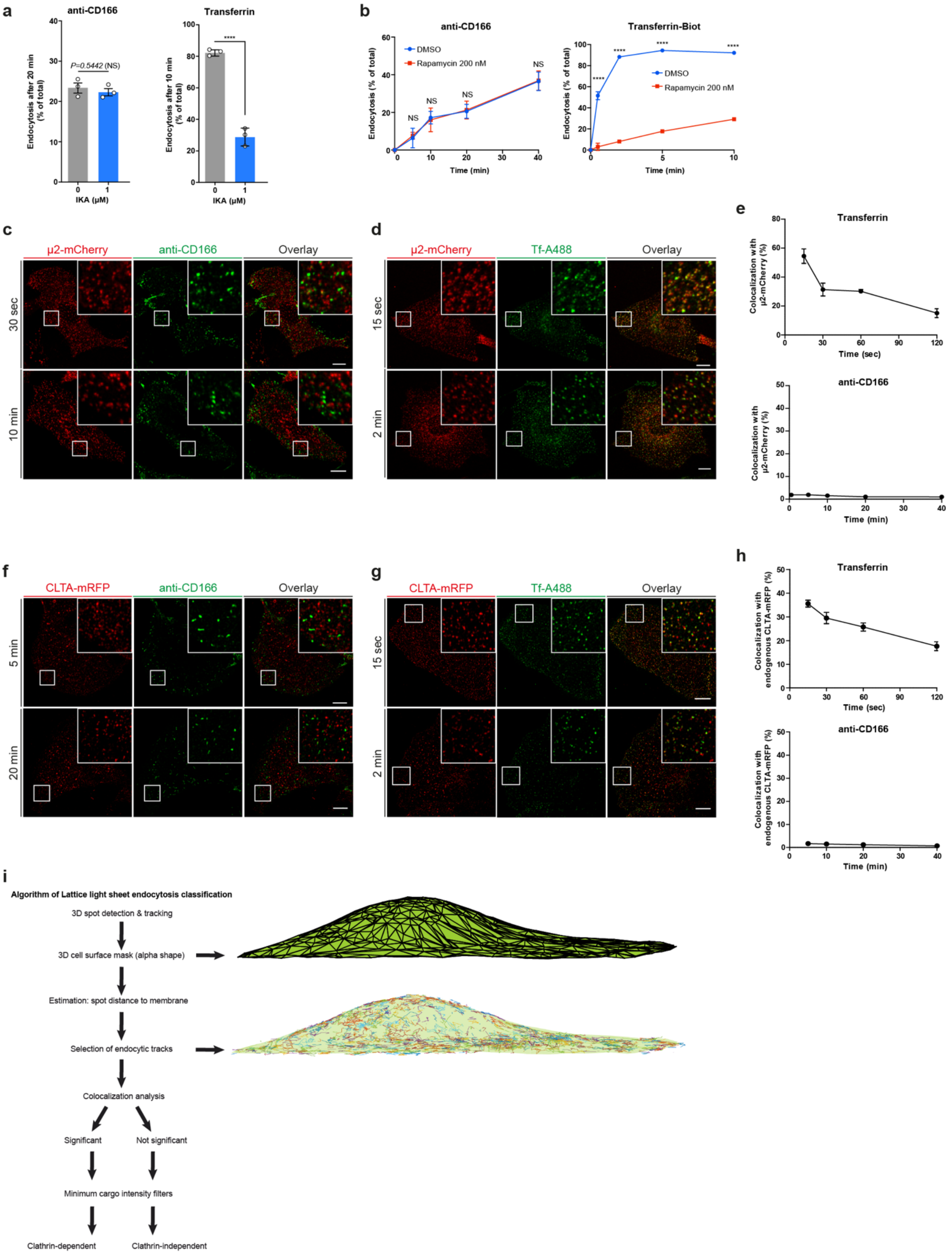

Supplementary Figure 2: Endocytosis of CD166 is clathrin- and dynamin-independent. a-b, Measure of anti-CD166 and transferrin (Tf) endocytosis by loss of surface assay in flow cytometry. **a,** HeLa cells pre-treated for 30 min with DMSO (control) or 1 μM ikarugamycin (IKA). Three independent experiments. NS, not significant. ****P < 0.0001 (Two-tailed unpaired T test with equal variances). Note the strong inhibition of Tf uptake (after 10 min) by ikarugamycin, while anti-CD166 antibody uptake remains constant (after 20 min). **b,** HeLaM cells engineered for Knocksideways of AP-

2 complex were treated for 10 min with DMSO (control) or 200 nM rapamycin. Three independent experiments. NS, not significant. ****$P < 0.0001$ (RM two-way ANOVA with Bonferroni's multiple comparison test). **c-e,** HeLa cells transiently expressing mCherry-tagged μ2-adaptin. **f-h,** Genome-edited U2OS cells expressing RFP-tagged clathrin light chain. After binding of anti-CD166 (**c,f**) or fluorescently labeled Tf (**d,g**) to cell surface on ice, cells were incubated at 37°C. **c,f,** Representative images of CD166 uptake after 30 sec and 10 min. **d,g,** Representative images of Tf uptake after 15 sec and 2 min. Graphs show quantifications of co-localization of anti-CD166 antibody or Tf with μ2-adaptin (**e**) or clathrin light chain (**h**) over time. Note the very low co-localization of CD166 with μ2-adaptin and clathrin light chain, by contrast with Tf. Number of cells: n = 5 (**c-e**); n = 15 (**f-h**). Three independent experiments (**c-h**). **i,** Algorithm of classification of endocytic events for CME versus CIE imaged by lattice light-sheet microscopy. Scale bars, 10 μm (**c,d,f,g**). Data are mean ± s.e.m. Source data are provided as a Source Data file.

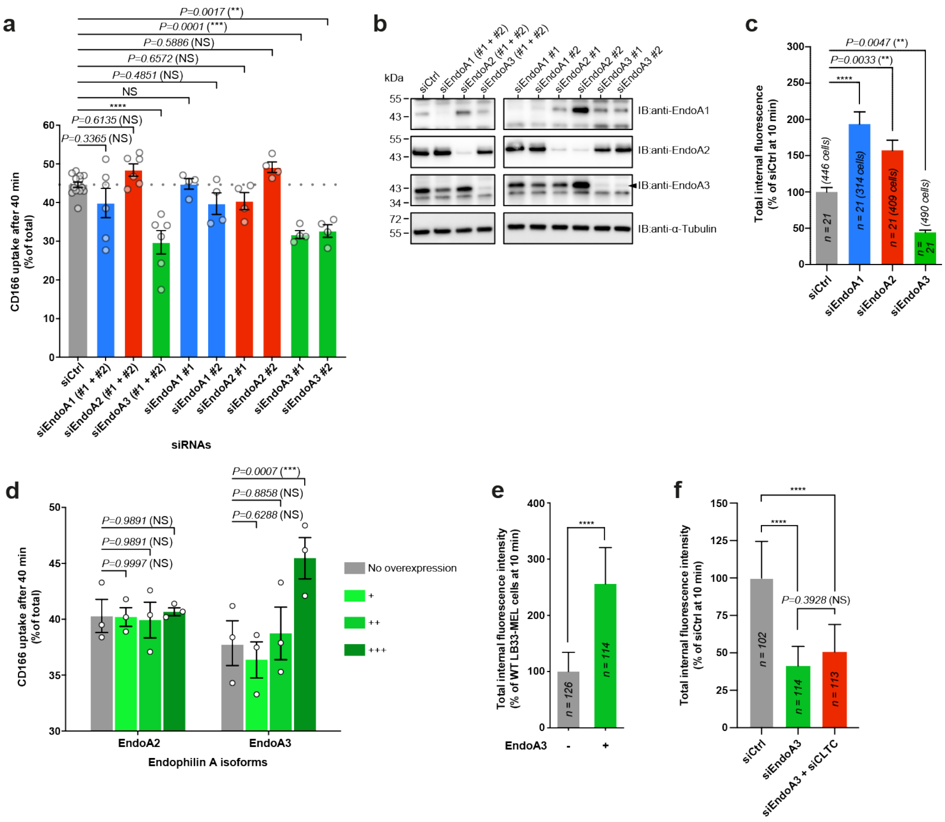

Supplementary Figure 3: Endophilin-A3 controls the clathrin-independent uptake of CD166. HeLa (**a,b,d,f**), U2OS (**c**) and LB33-MEL (**e**) cells. **a,** Anti-CD166 antibody endocytosis by loss of surface assay in flow cytometry on cells treated with control (siCtrl) or endoA1, A2 or A3 (siEndoA1, siEndoA2 or siEndoA3) siRNAs. siRNAs were used as pools of two sequences (#1 + #2) or as individual sequences (#1 or #2). Number of independent experiments: siCtrl, n = 14; pools of two siRNA sequences, n = 6; individual siRNA sequences, n = 4. NS, not significant. $**P < 0.01$, $***P < 0.001$, $****P < 0.0001$ (Ordinary one-way ANOVA with Dunnett's multiple comparison test and single pooled variance). **b,** Western blot analysis of endoA isoforms using isoform specific antibodies. Loading control, α-tubulin. Representative of three independent experiments. **c,** Continuous uptake of anti-CD166 antibody for 10 min in U2OS cells treated with siCtrl, siEndoA1, siEndoA2 or siEndoA3. Internal fluorescence quantified from confocal images and plotted as the relative percentage of siCtrl condition. n images per condition, three independent experiments. $**P < 0.01$, $***P < 0.001$ (Ordinary one-way ANOVA, with Tukey's multiple comparison test). **d,** Anti-CD166 antibody endocytosis by loss of surface assay in flow cytometry on cells transiently expressing endoA2-GFP or endoA3-GFP. Cells were sorted in four categories according to the expression level of GFP (no overexpression, +, ++ and +++). Gating strategy, Supplementary Figure 12b. Note the significant increase of anti-CD166 uptake upon strong endoA3 overexpression. n = 3 independent experiments. NS, not significant. $***P < 0.001$ (RM two-way ANOVA with Dunnett's multiple comparison test). **e,** Continuous uptake of anti-CD166 antibody for 10 min in wild-type (endoA3-) or stably expressing endoA3-GFP (endoA3+) LB33-MEL cells. Internal fluorescence quantified from confocal images and plotted as the relative percentage of wild-type (endoA3-) condition. n cells per condition, two independent experiments. $****P < 0.0001$ (Two-tailed Mann-Whitney test). **f,** Continuous uptake of anti-CD166 antibody for 10 min in cells treated with siCtrl, siEndoA3 or a combination siEndoA3 + siCLTC (clathrin heavy chain siRNAs). Internal fluorescence quantified from confocal images and plotted as the relative percentage of siCtrl condition. n cells per condition, three independent experiments. NS, not significant. $****P < 0.0001$ (Kruskal-Wallis test with Dunn's multiple comparison post hoc test, two-sided). Data are mean ± s.e.m. (**a,c,d**) or median ± 95% c.i. (**e,f**). Source data are provided as a Source Data file and in Supplementary Figure 11 (blots).

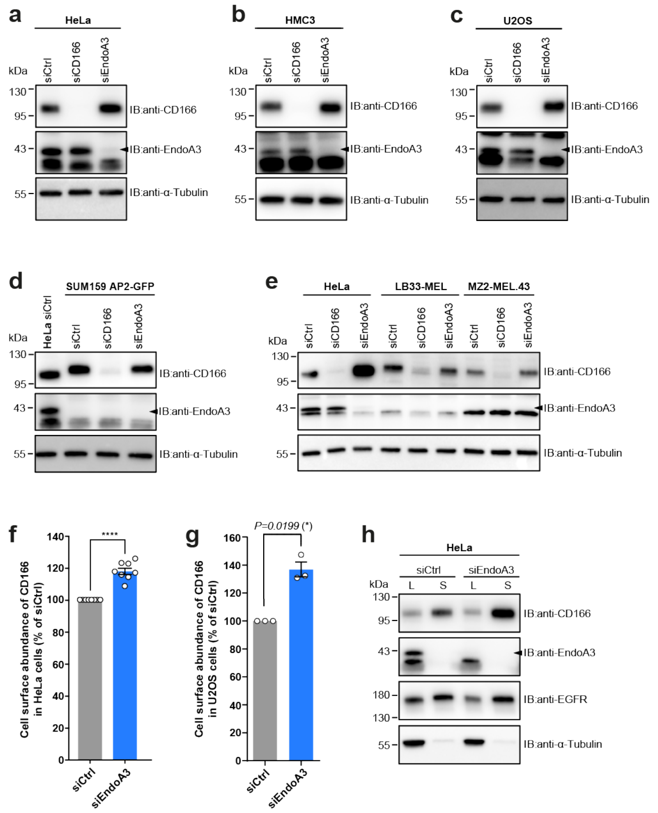

Supplementary Figure 4: CD166 expression and surface levels are dependent on endoA3. a-h, Various cell lines were transfected with negative control (siCtrl), CD166 (siCD166) or endoA3 (siEndoA3) siRNAs. **a-e,** Western blot analysis of cell lysates. **a,** HeLa (cervix adenocarcinoma), $n = 5$. **b,** HMC3 (immortalized microglia), $n = 2$. **c,** U2OS (osteosarcoma), $n = 3$. **d,** Genome-edited SUM159 AP2-GFP clone (breast carcinoma), $n = 2$. **e,** LB33-MEL and MZ2-MEL.43 (melanoma), $n = 2$. Representative of n independent experiments. Blots indicate that CD166 is expressed in all those cell lines. However, endoA3 is expressed in HeLa, HMC3 and U2OS cell lines, while not in SUM159 AP2-GFP, LB33-MEL and MZ2-MEL.43 (black arrows). Note the increased CD166 signal upon endoA3 depletion in the cell lines that express naturally endoA3 (**a-c**), while CD166 level remains unaffected in the others (**d,e**). **f-g,** Cell surface abundance of CD166 measured by flow cytometry in HeLa and U2OS cells, respectively, expressed as the percentage of control condition. Number of independent experiments: HeLa, $n = 8$; U2OS, $n = 3$. $*P < 0.05$, $****P < 0.0001$ (Two-tailed paired T test). Data are mean ± s.e.m. **h,** Western blot analysis of CD166 in total HeLa cell lysates (lane L) and cell surface fractions (lane S). Representative of three independent experiments. Anti-endoA3 antibody was used to show the efficiency of endoA3 depletion. EGFR and α-tubulin signals were used as a loading controls for cell surface fractions and total cell lysates, respectively. Note the increased CD166 signal in the cell surface fraction upon endoA3 depletion. Source data are provided as a Source Data file and in Supplementary Figure 11 (blots).

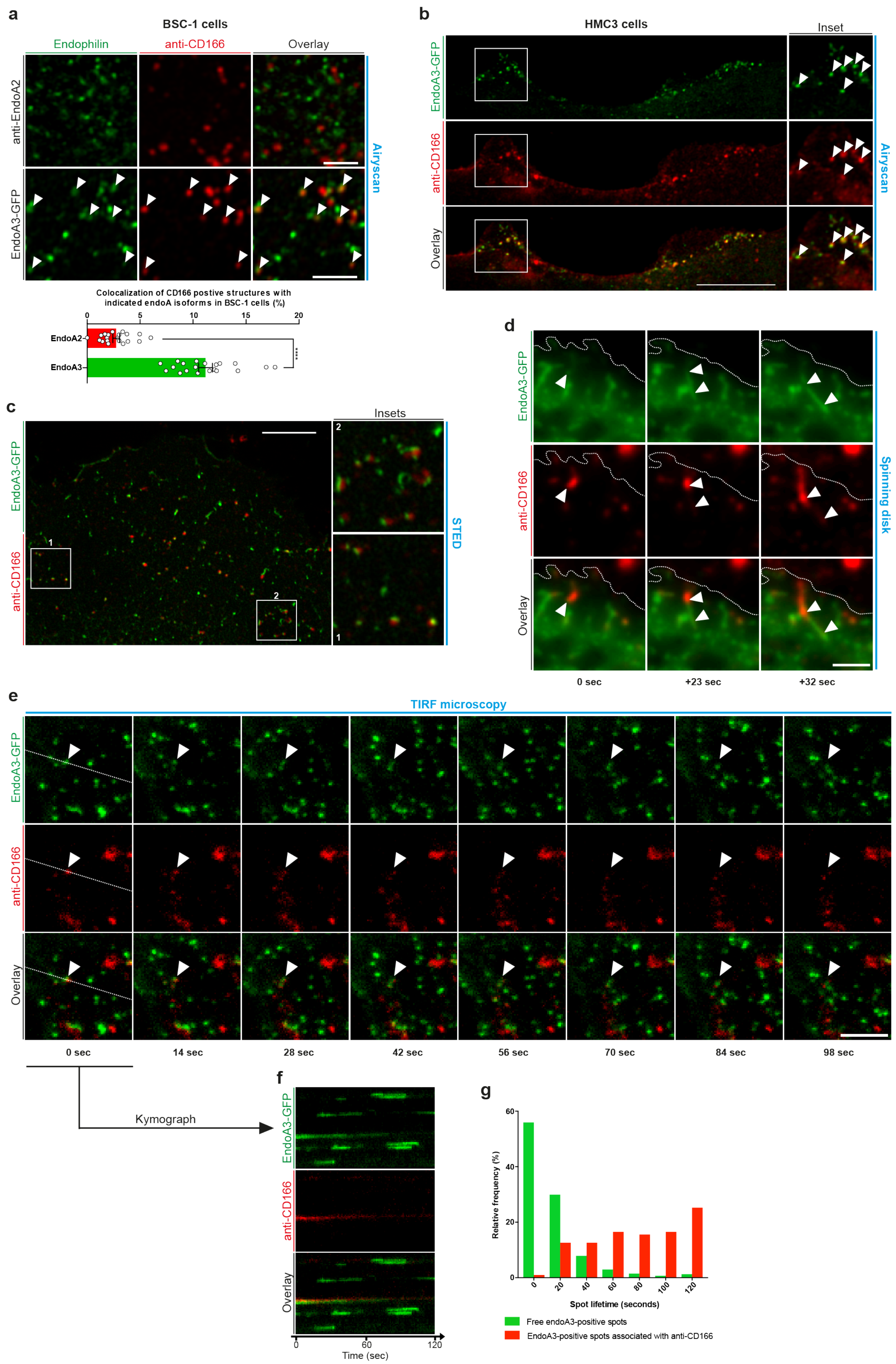

Supplementary Figure 5 (see next page)

Supplementary Figure 5: CD166 is endocytosed in endoA3-positive structures that are stabilized at plasma membrane upon association with the cargo. Anti-CD166 antibody uptake experiments in cell lines expressing GFP-tagged endoA3 (green signal): transient expression, BSC-1 (**a**) and HMC3 (**b**); stable expression, HeLa (**c-g**). Cells were continuously incubated with 5 µg ml^{-1} anti-CD166 antibody (red signal) at 37°C. **a-c**, After 10 min incubation, cells were fixed, permeabilized, labelled with fluorescently labeled secondary antibodies and observed in Airyscan confocal (**a,b**) or STED microscopy (**c**). Note the CD166-positive structures co-localizing with/wrapped in endoA3-GFP (white arrows). (**a**) $n = 20$ cells. Two independent experiments. ****$P < 0.0001$ (Two-tailed unpaired T test). (**b,c**) Representative of two independent experiments. **d-g,** Live spinning disk (**d**) and TIRF (**e-g**) microscopy on cells incubated with Alexa Fluor 647 or 555 labeled antibody, respectively (red signal). **d,** Formation of an endoA3-positive tubule emanating from cell surface in which CD166 is taken up over time (white arrows). The dotted line indicates cell surface. Two independent experiments. **e,** Time serie images from TIRF microscopy with kymograph (**f**) and plot of endoA3-GFP spot lifetime distribution (**g**). The dashed line indicates where the kymograph was obtained. Note the increased lifetime of endoA3-positive spots at cell surface upon association with anti-CD166 antibody (**e,** white arrow). This increase is statistically visible on the lifetime distribution graph (**g**; green bars: free endoA3-positive spots, $n = 19{,}239$ tracked spots; red bars: endoA3-positive spots associated with anti-CD166 antibody, $n = 103$ tracked spots; images from nine time series, three independent experiments). For full movies, see Supplementary Movies 3-5 and Movie 6 related to panels **d** and **e**, respectively. Scale bars, 10 µm (**b**), 5 µm (**c,e**), 2 µm (**a,d**). Source data are provided as a Source Data file.

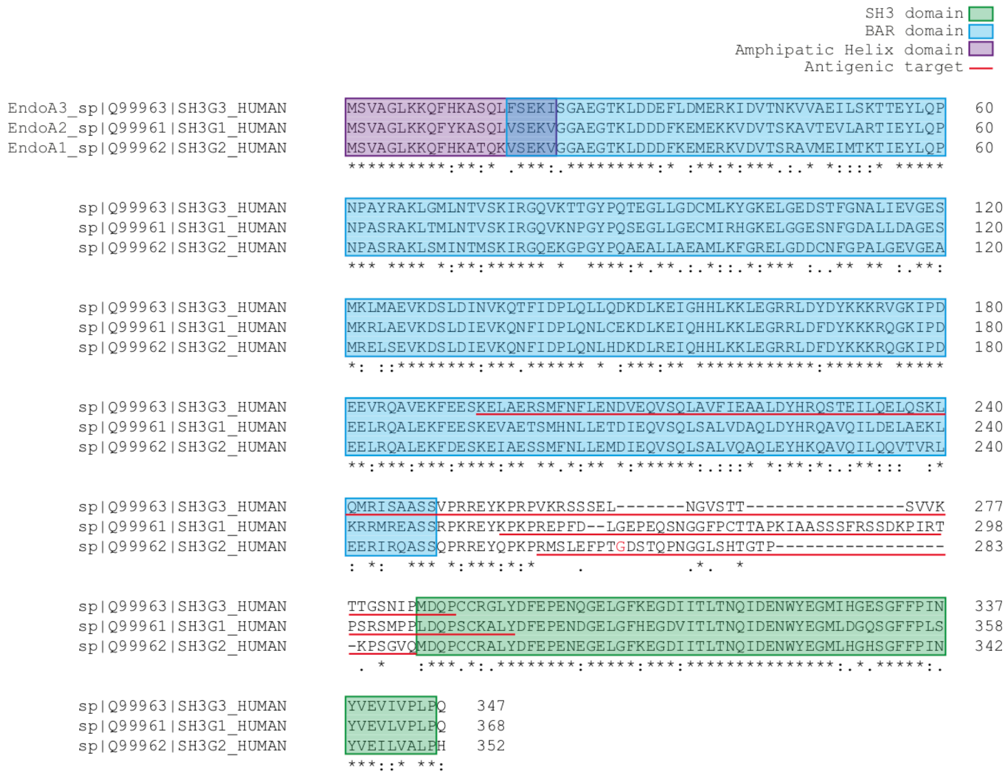

Supplementary Figure 6: Alignment of the three human endoA isoforms. Swissprot accession numbers: endoA1, Q99962; endoA2, Q99961; endoA3, Q99963. Color code: purple, N-terminal amphipathic helix; blue, BAR domain; green, SH3 domain. Note the high conservation of BAR and SH3 domains among the three isoforms, while the linker regions do not share any significant similarity. The residues underlined in red color in the linker region indicate the targets of the isoform specific antibodies used in this study.

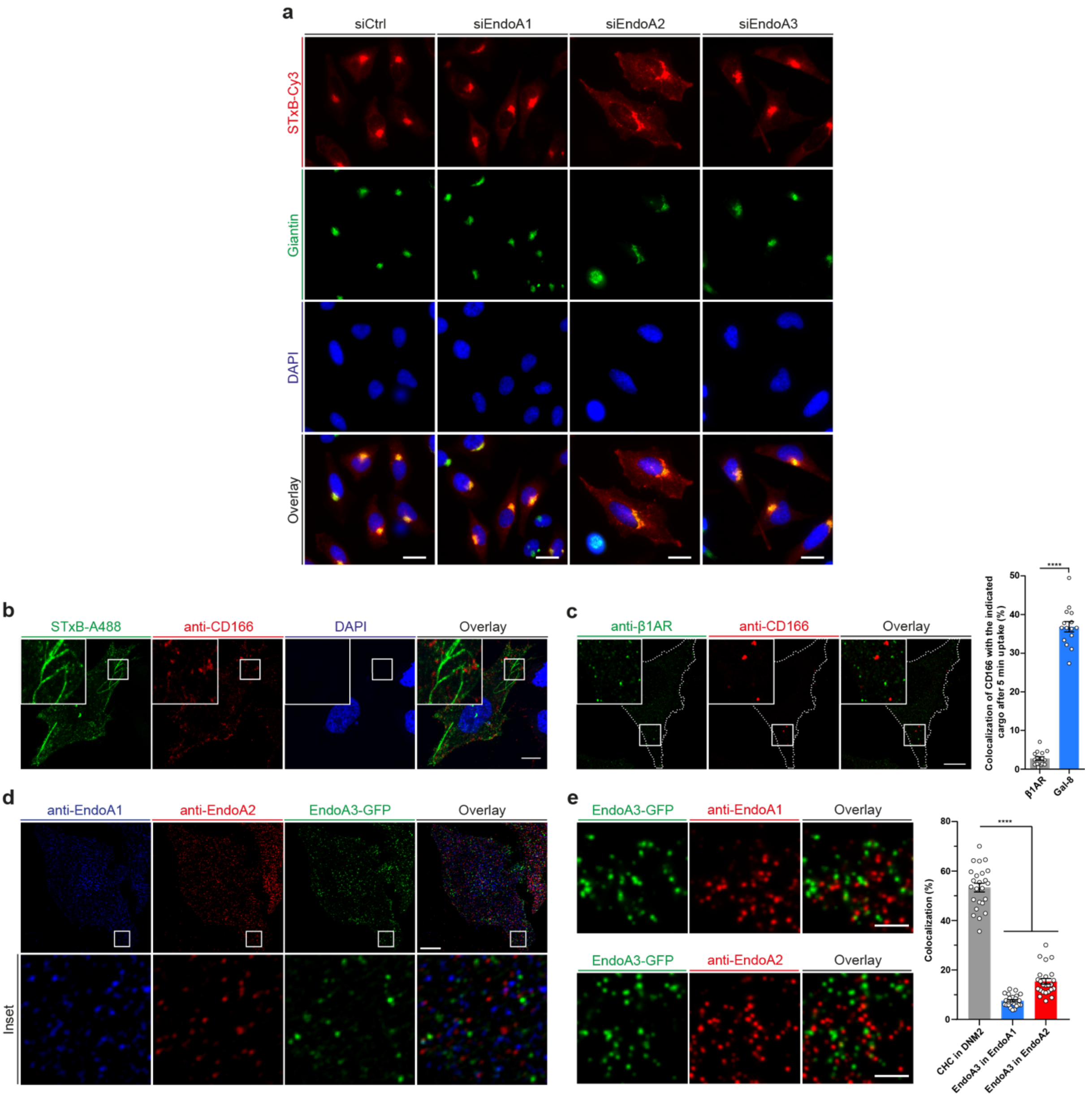

Supplementary Figure 7: CD166 does not co-localize with FEME cargoes, and endoA isoforms have distinct cellular localizations. a, HeLa cells treated with negative control (siCtrl) or endoA1, A2 or A3 isoforms (siEndoA1, siEndoA2 or siEndoA3) siRNAs were incubated for 45 min at 37°C with 50 nM STxB-Cy3. Representative images. Quantifications are presented in Figure 2e. **b,** ATP-depleted HeLa cells were incubated with 50 nM fluorescently labeled STxB (green channel) and CD166 was detected by immunofluorescence (red channel). Inset: white arrows indicate long tubular structures emanating from plasma membrane induced by STxB. Note that CD166 signal is absent from these STxB-positive tubules. **c,** Co-uptake experiments of CD166 and β1-adrenergic receptor. HeLa cells were co-incubated for 5 min with anti-CD166 antibody (5 µg ml^{-1}) and dobutamine (10 µM) at 37°C. β1-adrenergic receptor was immunodetected post-fixation. Note the localization of both markers in distinct endocytic compartments. Co-localization of Gal8 with CD166 from Figure 3a was used as a positive control. Quantifications of co-localization are shown on the graph (*n* = 15 cells, 3 independent experiments). ****P < 0.0001 (Two-tailed unpaired T test with Welch's correction). **d,** Confocal images of HeLa cells transiently expressing low level of endoA3-GFP (green). EndoA1 (red) and endoA2 (blue) were detected by immunofluorescence with isoform specific antibodies. Quantification of co-localization is in Figure 2h. For equivalent STED images, see Figure 2g. **e,** Co-localization analysis by TIRFM of endoA3 (endoA3-GFP, stable HeLa cell line) with endoA1 (anti-endoA1) and endoA2 (anti-endoA2). Co-localization of clathrin heavy chain with dynamin-2 was used as a positive control.

Quantifications are displayed on the graph (n = 24 cells). Three independent experiments. ****P < 0.0001 (One-way ANOVA with Tukey's multiple comparison test). Scale bars, 20 µm (**a**), 10 µm (**b-d**), 5 µm (**e**). Data are mean ± s.e.m. Source data are provided as a Source Data file.

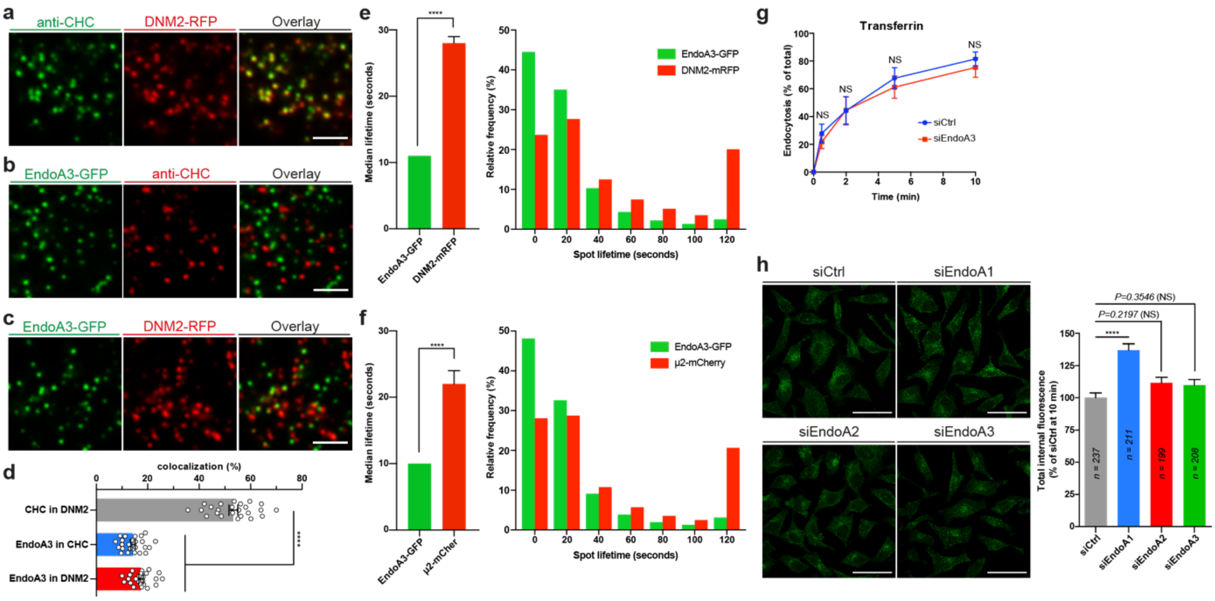

Supplementary Figure 8: Endophilin-A3 poorly co-localizes with CME markers and its depletion does not affect CME. a-f, Co-localization analyses of endoA3-GFP (stable HeLa cell line) with various CME markers. **a-d,** TIRF microscopy on fixed samples. **b,** Immunolabeling of endogenous clathrin heavy chain (anti-CHC). **c,** Transient expression of RFP-tagged dynamin-2 (DNM2-mRFP). Co-localization of clathrin heavy chain with dynamin-2 was used as a positive control (**a**). Quantifications of co-localization are displayed on the graph (**d**). For each condition: n = 24 cells. Three independent experiments. ****$P < 0.0001$ (Ordinary one-way ANOVA with Tukey's multiple comparison test and a single pooled variance). **e-f,** Quantifications of spot lifetime at plasma membrane from TIRF live cell imaging, related to Supplementary Movies 7 and 8. Graphs show the median lifetime (left panel) and the lifetime distribution (right panel) of spots for the indicated markers. **e,** Transient expression of DNM2-RFP. Number of spots: endoA3-GFP, n = 25,318 (9 images); DNM2-RFP, n = 10,745 (9 images). Three independent experiments. **f,** Transient expression of µ2-adaptin (µ2-mCherry). Number of spots: endoA3-GFP, n = 14,224 (6 images); µ2-mCherry, n = 5,632 (6 images). Three independent experiments. ****$P < 0.0001$ (Two-tailed Mann-Whitney test). Note the much shorter lifetime of endoA3 compared to dynamin-2 and µ2-adaptin. **g-h,** Uptake of the canonical CME cargo transferrin by HeLa cells treated with negative control (siCtrl) or endoA1, A2 or A3 (siEndoA1, siEndoA2 or siEndoA3) siRNAs. **g,** Measure of uptake over time by loss of surface assay in flow cytometry. Four independent experiments. NS, not significant (Mixed-effects model with Bonferroni's multiple comparison test). **h,** Continuous uptake of fluorescently labeled Tf performed for 10 min. Internal fluorescence was quantified from confocal images and plotted as the relative percentage of siCtrl condition. n cells per condition, three independent experiments. NS, not significant. ****$P < 0.0001$ (Ordinary one-way ANOVA with Bonferroni's multiple comparison test). Scale bar, 5 µm (**a,b,c**), 50 µm (**h**). Data are mean ± s.e.m. (**d,g,h**) or median ± 95% c.i. (**e,f**). Source data are provided as a Source Data file.

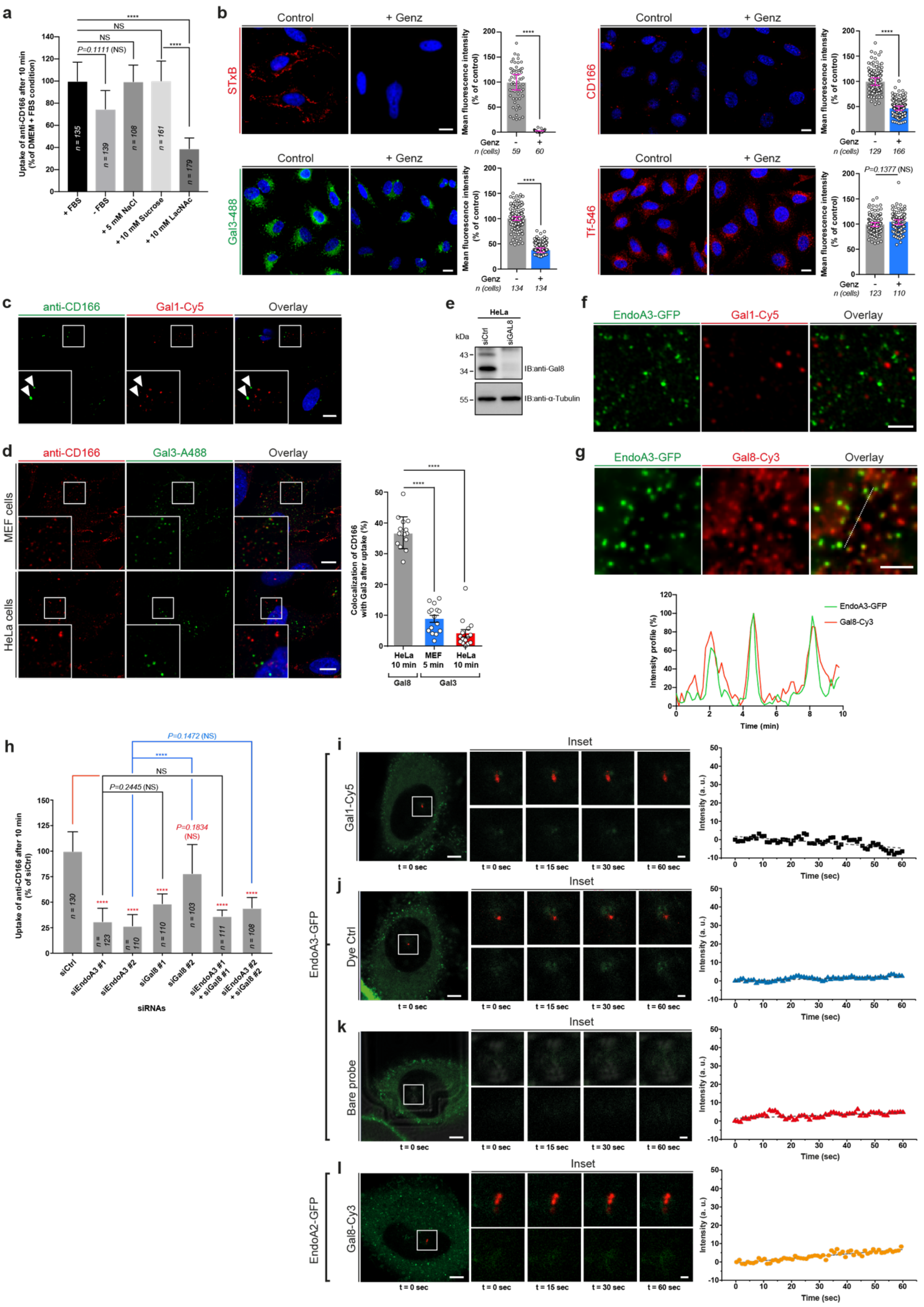

Supplementary Figure 9: Galectin-8 drives the uptake of CD166 and induces the recruitment of endophilin-A3 to endocytic sites at plasma membrane. HeLa (**a-l**) and MEF (**d**) cells. **a,** Effect of N-acetyl-D-lactosamine (LacNAc) on anti-CD166 antibody uptake. Cells incubated for 10 min with 5 µg

ml^{-1} anti-CD166 in indicated DMEM media: with serum (+FBS), without serum (-FBS), or without serum and supplemented with 5 mM NaCl, 10 mM sucrose or 10 mM LacNAc. Internal fluorescence quantified from confocal images and plotted as the relative percentage of +FBS condition. *n* cells per condition, four independent experiments. NS, not significant. ****$P < 0.0001$ (Kruskal-Wallis test with Dunn's multiple comparison post hoc test, two-sided). Note the significant decrease of anti-CD166 uptake in the presence of the galectin competitor LacNAc. **b,** Effect of depletion of glycosphingolipids on anti-CD166 uptake. Cells treated with 5 µM glucosylceramide synthase inhibitor Genz-123346 for 2 days were incubated with 5 µg ml^{-1} anti-CD166 antibody (top right panel), 200 nM fluorescent Galectin-3 (Gal3-A488, bottom left panel) or 10 µg ml^{-1} fluorescent transferrin (Tf-A546, bottom right panel) for 10 min at 37°C. Internal fluorescence quantified from confocal images. Note the significant decrease of anti-CD166 uptake upon Genz treatment, similar to Gal3. Tf uptake was not affected. Efficiency of glycosphingolipid depletion was verified with binding of STxB at cell surface (top left panel). *n* cells per condition, two independent experiments. NS, not significant. ****$P < 0.0001$ (Two-tailed Mann-Whitney test). **c,** Co-incubation of cells with 5 µg ml^{-1} anti-CD166 antibody and 50 nM fluorescent Galectin-1 (Gal1) for 10 min at 37°C. Note the poor co-localization of CD166 with Gal1 (white arrows). Gal8 condition, Figure 3a. Three independent experiments. **d,** MEF and HeLa cells incubated with 5 µg ml^{-1} anti-CD166 and 200 nM fluorescent Gal3 for 5 and 10 min at 37°C, respectively. Graph, quantifications of co-localization ($n = 15$ cells). Three independent experiments. Data statistically compared to co-localization with Gal8 (Figure 3a). ****$P < 0.0001$ (Ordinary one-way ANOVA with Bonferroni's multiple comparison test). Note the poor co-localization of CD166 with Gal3. **e,** Western blot analysis on HeLa cells treated with negative control siRNA (siCtrl) or a smartpool of four siRNAs against endogenous Gal8 (siGAL8). Anti-Gal8 blot shows the efficiency of Gal8 depletion. Loading control, α-tubulin. Related to Figure 3b. Three independent experiments. **f**, Cells stably expressing endoA3-GFP incubated with 50 nM fluorescent Gal1 for 10 min at 37°C. Note the poor co-localization. Gal8 condition, see Figure 3c. Three independent experiments. **g,** TIRFM analysis of co-localization between endoA3 and Gal8. Cells stably expressing endoA3-GFP continuously incubated with 100 nM Gal8-Cy3. White arrows, co-localization spots. Dashed line shows, position of intensity profile (graph). Three independent experiments. **h,** Cells treated with control siRNA (siCtrl), individual siRNA sequences against endoA3 (siEndoA3 #1 and #2), individual sequences against Gal8 (siGal8 #1 and #2), or combination of sequences (siEndoA3 #1 + siGal8 #1 or siEndoA3 #2 + siGal8 #2), were incubated for 10 min at 37°C with 5 mg ml^{-1} anti-CD166 in serum-free media. Internal fluorescence quantified from confocal images and plotted as the relative percentage of control condition. *n* cells per condition, three independent experiments. NS, not significant. ****$P < 0.0001$ (Kruskal-Wallis test with Dunn's multiple comparison post hoc test, two-sided). Note that combination of endoA3 and Gal8 depletion did not further reduce CD166 uptake. **i-l,** Experiments with Fluid-FM coupled to confocal setup, related to Figure 3d-f. **i-k,** EndoA3-GFP expressing cells approached with Gal1-coated nanoparticle (**i**, $n = 10$), fluorescent nanoparticle without protein coating (dye control, **j**, $n = 5$), or bare AFM probe without nanoparticle (**k**, $n = 5$). *n* indicates independent measurements. **l,** EndoA2-GFP expressing cells approached with Gal8-coated nanoparticle ($n = 10$ independent measurements). Left: overview of cells approached by functionalized gold bead trapped on the Fluid-FM probe (t = 0 sec, initial cell exposure). Insets: area surrounding beads at indicated time points. Green channel, endoA3-GFP (**i-k**) or endoA2-GFP (**l**). Red channel, Gal8, Gal1 or dye control. For the bare probe control, an overlay between the transmitted light channel and the endoA3-GFP channel is used to show the positioning of the Fluid-FM probe on the cell. Right: corresponding plots of green fluorescence intensity around the functionalized bead as a function of time. Scale bars, 2 µm (**f**), 5 µm (**g,i-l**), 10 µm (**b-d**). Data are median ± 95% c.i. (**a,h**) or mean ± s.e.m. (**b,d**). Source data are provided as a Source Data file and in Supplementary Figure 11 (blots).

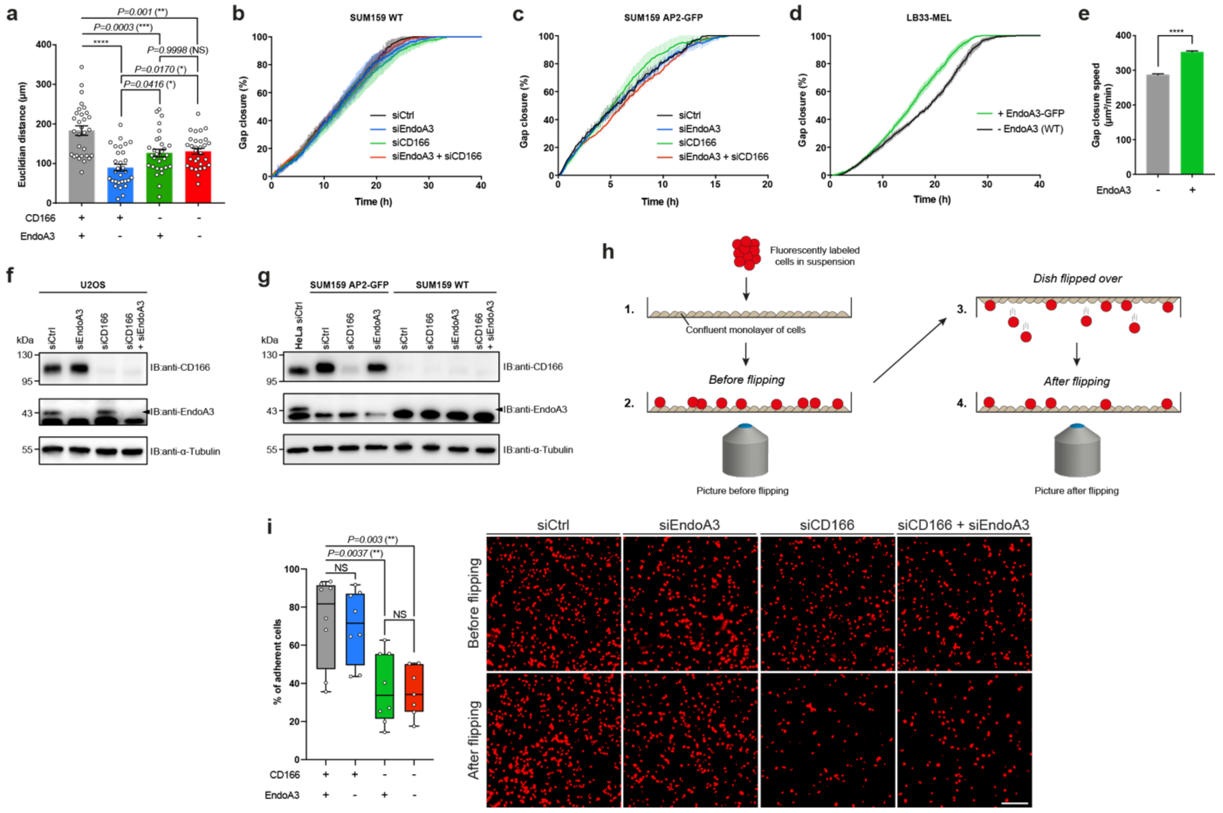

Supplementary Figure 10: Modulation of CD166 cell surface abundance by endoA3-dependent endocytosis affects cancer cell adhesive and migratory properties (related to Figure 4). U2OS (**a,f**), wild-type SUM159 (**b,g**), or genome-edited SUM159 AP2-GFP (**c,g**) treated with negative control (siCtrl, grey), endoA3 (siEndoA3, blue), CD166 (siCD166, green) siRNAs or a combination (red). LB33-MEL (**d**) was used as wild-type (- endoA3) or stably expressing endoA3-GFP (+ endoA3-GFP). **a,** Euclidian distance, related to wound healing assays in Figure 4a-e. NS, not significant. *$P < 0.05$, **$P < 0.01$, ***$P < 0.001$, ****$P < 0.0001$ (Ordinary one-way ANOVA, with Tukey's multiple comparison test). **b-e,** Wound healing assays on other cancer cell lines. **b,** Wild-type SUM159. See Supplementary Movie 15 ($n = 4$, two independent experiments). **c,** Genome-edited SUM159 AP2-GFP. See Supplementary Movie 16 ($n = 2$ technical replicates, representative of two independent experiments). Note that siRNA transfections do not modify gap closure for SUM159 WT and SUM159 AP2-GFP. **d,e,** LB33-MEL. See Supplementary Movie 17. Average gap closure speed (**e**) was extracted from linear regressions fitted to curves in **d**. Note that expression of endoA3 in LB33-MEL significantly increases gap closure speed ($n = 6$, three independent experiments). ****$P < 0.0001$ (Two-tailed unpaired T test). **f,g,** Western blot analysis of cell lysates: U2OS (**f**, $n = 2$), SUM159 AP2-GFP and SUM159 wild-type (**g**, $n = 2$). Anti-CD166 and anti-endoA3 antibodies were used to show the efficiency of CD166 and endoA3 depletion, respectively, or their absence in the cell lines. Loading control, α-tubulin. Note that U2OS are CD166+/endoA3+ (similar to HeLa cells), while SUM159 AP2-GFP are CD166+/endoA3- and SUM159 wild-type are CD166-/endoA3-. Representative of n independent experiments. **h,** Scheme of cell-cell adhesion flipping assay for measurement of intercellular adhesion. (1) Cells in suspension are labeled with a fluorophore (red) and (2) sedimented for 10 min in a dish containing a confluent monolayer of unlabeled cells. A first picture is then taken, before flipping. (3) The dish is flipped over for 15 min, allowing suspension cells that did not adhere to the confluent monolayer to detach. (4) The dish is then returned to its original position, and a second picture is taken, after flipping. The pictures taken before and after flipping allow to calculate the percentage of cells in suspension that adhered to the confluent monolayer. **i,** Quantification of adhesiveness of cells in suspension treated with siCtrl (grey), siEndoA3 (blue), siCD166 (green) or a combination (red) to a confluent monolayer of untreated wild-type cells, using the flipping assay. Quantifications, and representative images before and after flipping are shown (siCtrl, $n = 8$; siEndoA3, $n = 8$; siCD166, $n = 8$; siCD166 + siEndoA3, $n = 7$). Three independent experiments. NS, not significant. **$P < 0.01$

(Ordinary one-way ANOVA with Bonferroni's multiple comparison test). Note the decreased adhesion upon CD166 depletion. Scale bar, 200 µm (**i**). Data are mean ± s.e.m. (**a**,**b**,**d**,**e**) or mean ± range (**c**). Box plots (**i**) show median (–), mean (+), 25th and 75th percentiles (boxes) and data range from min to max (whiskers). Source data are provided as a Source Data file and in Supplementary Figure 11 (blots).

Figure 1

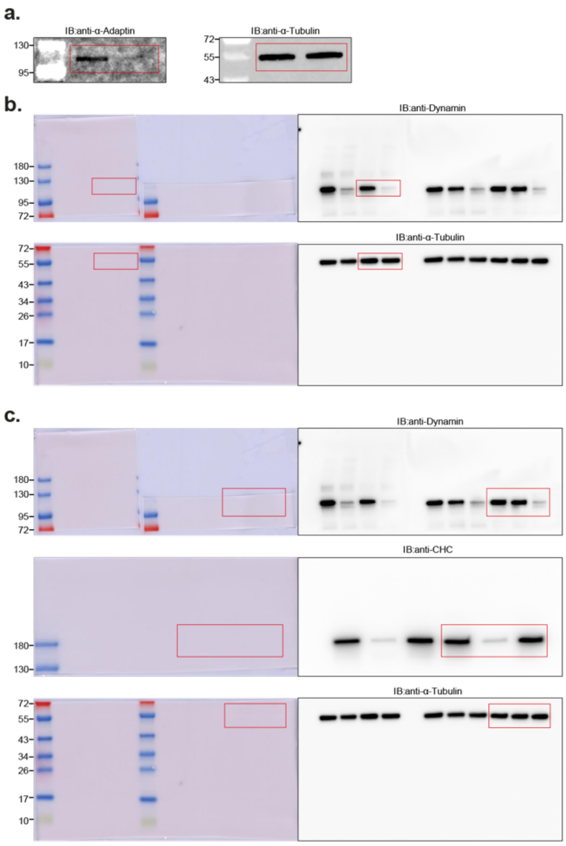

Figure 2

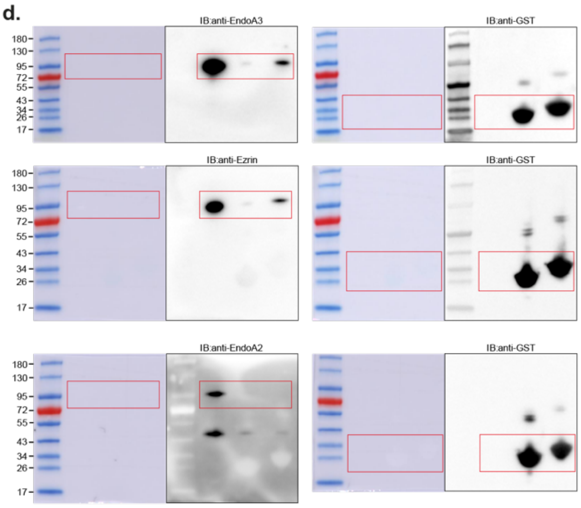

Supplementary Figure 1

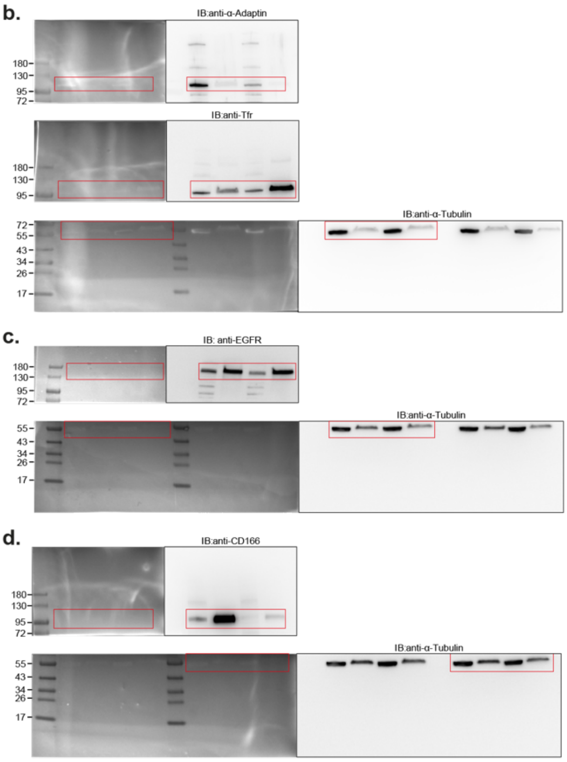

Supplementary Figure 11 (part 1 of 3).

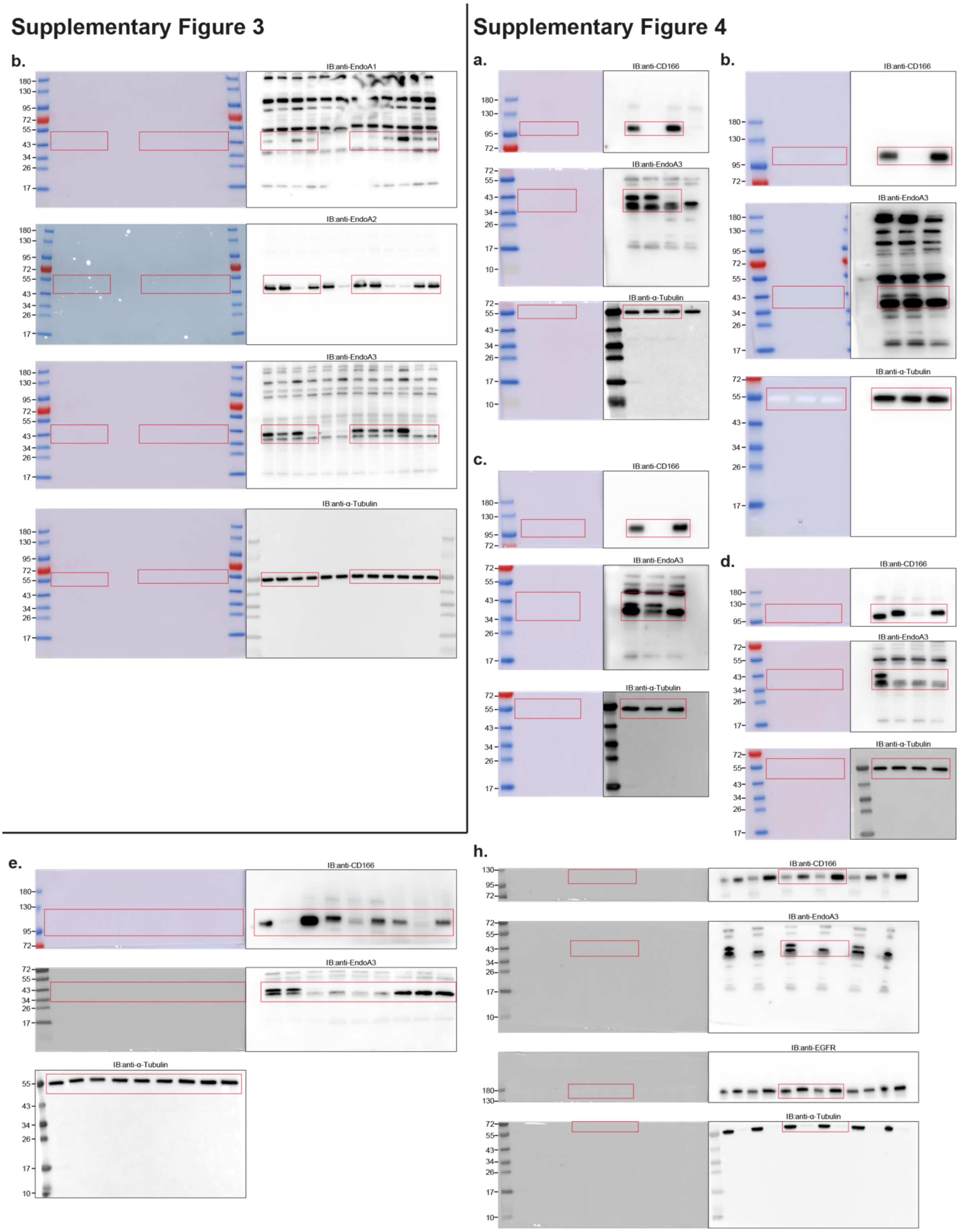

Supplementary Figure 11 (part 2 of 3).

Supplementary Figure 9

e.

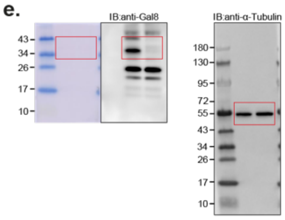

Supplementary Figure 10

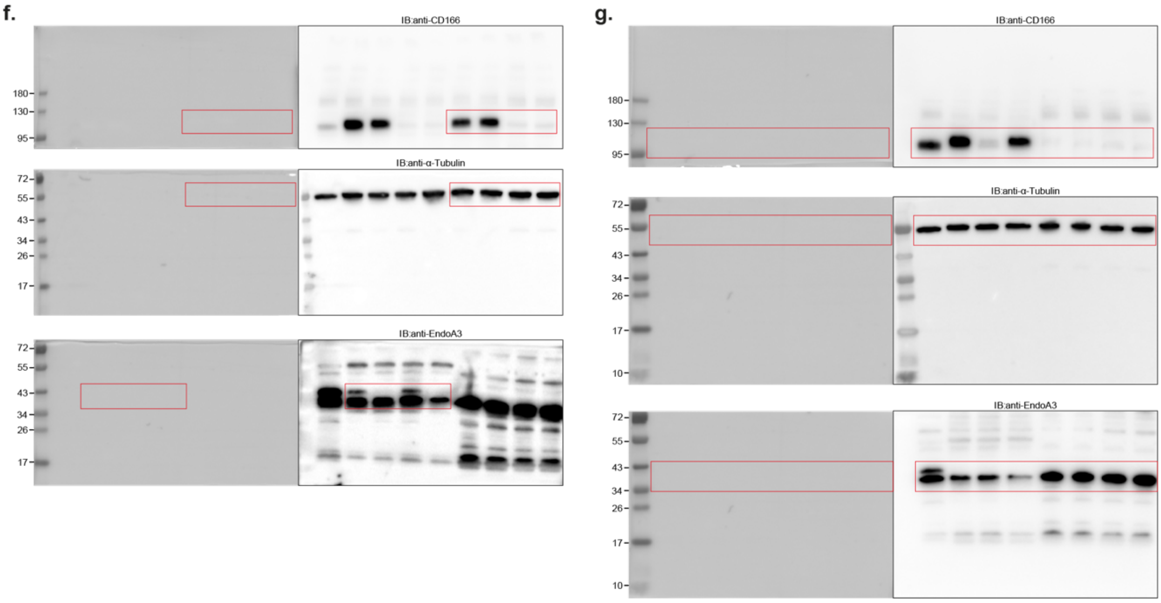

Supplementary Figure 11 (part 3 of 3): Uncropped Western blot images with molecular weight markers. The corresponding figures and supplementary figures in which cropped images were included (red marks) are indicated on each uncropped image.

a

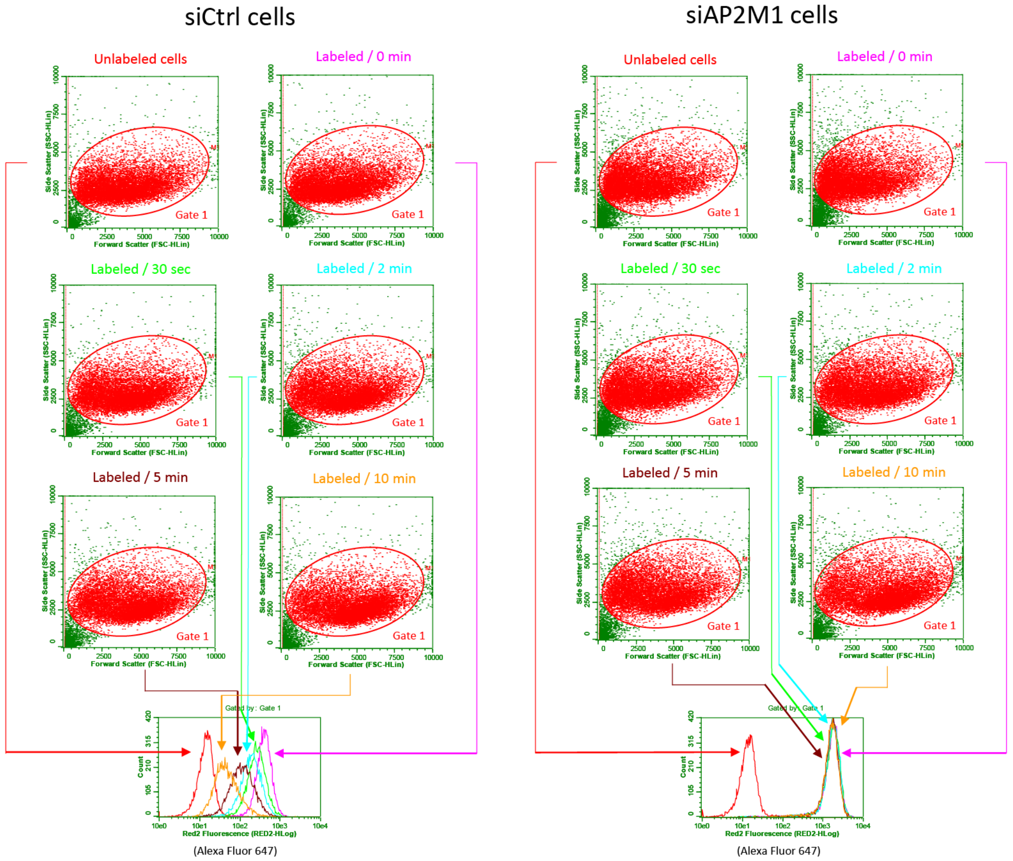

		Number of cells in Gate 1
siCtrl	Unlabelled	10000
	Labeled / 0min	10000
	Labeled / 30sec	10000
	Labeled / 2min	10000
	Labeled / 5min	10000
	Labeled / 10min	10000

		Number of cells in Gate 1
siAP2M1	Unlabelled	10000
	Labeled / 0min	10000
	Labeled / 30sec	10000
	Labeled / 2min	10000
	Labeled / 5min	10000
	Labeled / 10min	10000

Median fluorescence intensity
measured at cell surface at different
time points after placing cells at 37°C

Calculation of % of endocytosis
at each time point
(0% of endocytosis is the 0 min time point
= 100% of the cargo at cell surface)

Supplementary Figure 12 (part 1 of 2).

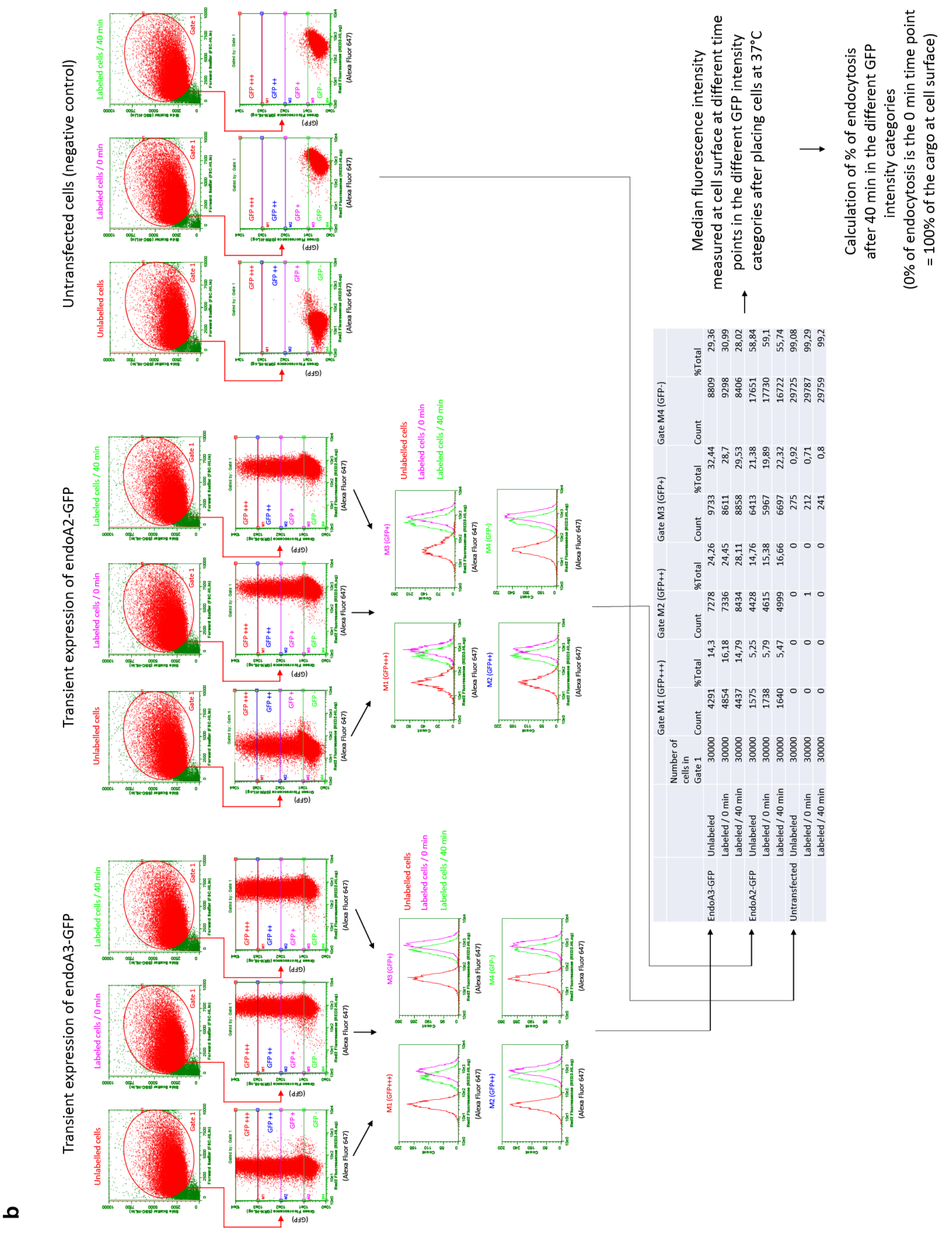

		Number of cells in Gate 1	Gate M1 (GFP+++) Count	Gate M1 (GFP+++) %Total	Gate M2 (GFP++) Count	Gate M2 (GFP++) %Total	Gate M3 (GFP+) Count	Gate M3 (GFP+) %Total	Gate M4 (GFP-) Count	Gate M4 (GFP-) %Total
EndoA3-GFP	Unlabeled	30000	4291	14,3	7278	24,26	9733	32,44	8809	29,36
	Labeled / 0 min	30000	4854	16,18	7336	24,45	8611	28,7	9298	30,99
	Labeled / 40 min	30000	4437	14,79	8434	28,11	8858	29,53	8406	28,02
EndoA2-GFP	Unlabeled	30000	1575	5,25	4428	14,76	6413	21,38	17651	58,84
	Labeled / 0 min	30000	1738	5,79	4615	15,38	5967	19,89	17730	59,1
	Labeled / 40 min	30000	1640	5,47	4999	16,66	6697	22,32	16722	55,74
Untransfected	Unlabeled	30000	0	0	0	0	275	0,92	29725	99,08
	Labeled / 0 min	30000	0	0	1	0	212	0,71	29787	99,29
	Labeled / 40 min	30000	0	0	0	0	241	0,8	29759	99,2

Supplementary Figure 12 (part 2 of 2): Gating strategies of flow cytometry experiments. a, Gating strategy applied for cell surface staining and endocytosis measurements by loss of surface assays. This strategy was the same for all cargoes (CD166, Tf) in Figure 1a,b and Supplementary Figures 2a,b, 3a, 4f,g, 8g. Flow cytometry was always performed on 10,000 events representing at least 50% of the total cell population (no tracking of specific subpopulations). This figure shows a representative experiment for Tf uptake presented in Figure 1a. **b,** Gating strategy applied for CD166 endocytosis measurements by loss of surface assays upon increasing endoA3 expression levels, related to Supplementary Figure 3d. Flow cytometry was performed on 10,000 or 30,000 events representing at least 50% of the total cell population (Gate 1). Secondary gating was applied to divide the cell population selected in Gate 1 into four categories with differential GFP expression levels: Gate M1 (high level, GFP+++); Gate M2 (medium level, GFP++); Gate M3 (low level, GFP+); Gate M4 (no expression, GFP-). Of note, untransfected cells were used as a negative control and show that more than 99% of cells fall into Gate M4. This figure shows a representative experiment of Supplementary Figure 3d.

Supplementary Methods

Recombinant protein production

Human Gal8 was amplified from a DNA template encoding the NM_201544.2 version and cloned in the pHis-Parallel 2 plasmid with a N-terminal 6xHis-tag (forward primer with NdeI restriction site: 5'-GGAATTCCATATGTCGTACTACCATCACCATCACCATCACGCCATGTTGTCCTTAAACAAC CTACAG-3'; reverse primer with NotI restriction site: 5'-ATAAGAATGCGGCCGCCTACCAGCTCC TTACTTCCAGTAAGTGG-3'). The construct was verified by sequencing. All galectins used in this study – Gal3-His[1], Gal1-His[2] and His-Gal8 in pHis-Parallel 2 – were expressed at 20°C overnight in Rossetta2-pLysS *E. coli* (Novagen) in LB media, supplemented with 60 µM IPTG. Bacterial pellets were resuspended and sonicated in: PBS pH 7.3 for Gal3; PBS pH 7.3, 10% glycerol, 5 mM beta-mercaptoethanol for Gal1; 25 mM HEPES pH 7.3, 300 mM NaCl, 10 mM imidazole, 10% glycerol for Gal8. The Gal8 lysate was adjusted to 5% (w/v) ELUGENT (Merck), kept agitating for 30 min at 4°C. All lysates were cleared by centrifugation at 75,000xg for 1 h. 95% purity by Coomassie staining was achieved using Cobalt-resin (Pierce) affinity chromatography. Cobalt-resin was washed with resuspension buffer containing 20 mM imidazole for Gal1 and Gal3, and resuspension buffer containing 1% ELUGENT for Gal8. Elution was performed with the following buffers: resuspension buffer containing 300 mM imidazole for Gal1 and Gal3; resuspension buffer containing 20 mM trehalose, 0.2% (w/v) PEG8000 and 300 mM imidazole for Gal8. Gel filtration (HiLoad Superdex 75 16/600) was performed in elution buffer without imidazole. Galectin aliquots were snap frozen in liquid nitrogen and stored at -80°C.

Recombinant protein and antibody labeling

For light microscopy experiments, anti-CD166 antibody (Bio-Rad, MCA1926) was labeled with Alexa Fluor 555 or 647 NHS-ester dyes (Thermo Fisher Scientific), according to manufacturers' recommendations. For lattice light-sheet microscopy (LLSM), anti-CD166 antibody or Transferrin were labeled with ATTO 647N NHS-ester dye (ATTO-TEC GmbH), according to manufacturers' recommendations. Briefly, the protein was suspended in a PBS solution, supplemented with 100 mM or 10 mM sodium bicarbonate pH 9 (for Alexa and ATTO dyes, respectively). A 4- to 10-fold molar excess of dye (resuspended and conserved in DMSO) was added to the protein solution. The reaction mix was protected from light and incubated for 30 min to 1h at room temperature, or over-night at 4°C under mild agitation. The coupling reaction was stopped by the addition of Tris buffer pH 8.0 at a final concentration of 50 mM. Free dye was then removed by gel filtration on PD SpinTrap G-25 columns (GE Healthcare), and the labeled protein was recovered in PBS.
Galectins were coupled to mono-amine-reactive (NHS-ester) Cy3, Cy5 (GE Healthcare) or Alexa-488 in final purification buffer supplemented with 10 mM lactose for 30 min at room temperature, using a molar ratio of 1:4 (protein/dye). The mixture was purified in the same buffer without lactose and a PD-10 column (GE Healthcare). Labelling efficiency was 0.9 (Gal1), 1.3 (Gal3) and 1.0 (Gal8).

Isolation of cell surface proteins and quantitative mass spectrometry

Isolation of cell surface proteins. 48 h after transfection with AllStars Negative control or µ2-adaptin siRNAs, HeLa cells were transferred in 15 cm diameter cell culture dishes for an additional 24 h in order to obtain two 90-95% confluent dishes per condition. Surface proteins were then recovered using the Pierce Cell Surface Protein Isolation Kit (Thermo Fisher Scientific). In brief, after washes with ice-cold PBS^{++}, cells were incubated for 30 min at 4°C with 0.4 mM EZ-Link Sulfo-NHS-SS-Biotin in PBS^{++}. Cells were then washed with ice-cold PBS++, residual biotin compound was quenched with the kit quenching solution for 30 min, and two additional washes with ice-cold TBS (25 mM Tris-HCl pH 7.2, 150 mM NaCl) were performed. Cells were then scrapped and sonicated on ice in lysis buffer (10 mM Tris-HCl pH 7.5, 150 mM NaCl, 0.5% SDS, 1 mM EDTA, 1 mM EGTA, 2% NP-40, 10 mM

iodoacetamide, 1 mM PMSF and protease inhibitor cocktail). After incubation of ice for 30 min with vortexing from time to time, lysates were clarified at 2,500xg for 5 min at 4°C. Clarified lysates were then incubated on NeutrAvidin agarose resin over-night at 4°C on a rotating wheel. Flow-through was discarded, after which resin was washed twice with lysis buffer, twice with high salt lysis buffer (500 mM NaCl) and once with a Tris buffer (10 mM Tris-HCl pH 7.5, 1 mM EGTA, 1 mM EDTA). Proteins were then eluted by heating for 5 min at 95°C in elution buffer (100 mM Tris-HCl pH 7.6, 2% SDS, 100 mM DTT).

Preparation of peptides by FASP (Filter-Aided Sample Preparation). Tryptic digestion was performed on a 30 kDa cut-off filter, according to FASP protocol[3]. Briefly, 60-100 µg eluted proteins were loaded on filter units and centrifuged several times for 10-15 min at 14,000xg. Lysis buffer was progressively replaced by 100 mM Tris-HCl pH 8.5 containing 8 M urea. A second alkylation step was added here: filter units were incubated with 100 mM Tris-HCl pH 8.5, 8 M urea, 50 mM iodoacetamide for 20 min. Urea buffer was then progressively replaced by 100 mM triethyl ammonium bicarbonate buffer (TEAB, pH 8.5). PNGase F (New England Biolabs) treatment was performed on the filter unit for 4h at 37°C. Subsequent tryptic digestion (Promega) was performed over-night in 100 mM TEAB on the filter unit at an enzyme to protein ratio of 1:20. Peptides were eluted by centrifugation, and a last wash of the filter unit was performed with 0.5 M NaCl to recover non-eluted peptides. Peptides were quantified here by UV spectrometry at 280 nm.

iTraq labeling and proteomic analysis. 20 µg of peptides of negative control and µ2-adaptin depleted conditions were vacuum-dried and iTraq 4-plex labelling was performed for 1h using the manufacturer's kit and protocol (Applied Biosystems). Two technical replicates of control condition were produced by labeling with 114 and 115 Da iTraq reporters. Similarly, two technical replicates of µ2-adaptin-depleted condition were produced by labeling with 116 and 117 Da iTraq reporters. 2 µg of labeled peptides were resuspended in 2% (v/v) acetonitrile and 0.5% (v/v) formic acid, and desalted on a C18 pre-column (Acclaim PepMap 100 C18, 2 cm, 100 µm, Thermo Fisher Scientific). Eluted peptides were immediately subjected to reverse phase chromatography on a C18 analytical column (Acclaim PepMap 100 C18, 75 µm id x 25 cm, Thermo Fisher Scientific) for 150 min with a linear gradient from 5 to 35% (v/v) acetonitrile in 0.1% (v/v) formic acid. The iTraq-labeled peptides were analyzed by electrospray ionization on a TripleTOF 5600^{+} System (AB Sciex) mass spectrometer.

Data analysis. Mass spectrometry data were analyzed with ProteinPilot Software 4.5 (AB Sciex). MS/MS spectra were analyzed thanks to reviewed human Swiss-Prot database (2019-01-15). The "iTRAQ 4plex peptide labelled" sample type and "a biological modification ID focus" were selected in the analysis method. Trypsin was selected as the digestion enzyme, with cysteine alkylation by iodoacetamide as modification. Data were normalized by ProteinPilot algorithm. All reported data were based on 99% confidence for protein identification as determined by ProteinPilot (ProtScore ≥ 2). The confidence in protein identification was expressed as the "Unused ProtScore", which only considers peptides from spectra that have not already been used to identify higher scoring proteins. Proteins were further annotated based on GO annotations found in Swiss-Prot database. Relative quantification of cell surface proteins was obtained thanks to the iTraq labelling. In order to calculate ratios – corresponding to the fold change of abundance of a given protein between two samples – we used the sample labeled with the 114 Da reporter (negative control) as the reference. Each ratio is accompanied by a p-value calculated by ProteinPilot software, that reflects the confidence in the fact that the ratio is significantly different of unity. P-values take into account the quality of identification (*i.e.* number of peptides and confidence of identification of peptides). Ratios of relative abundance of each protein in the different conditions and their p-value were presented as Volcano plots (Supplementary Figure 1a).

Quantification of gene expression by qPCR

RNA extraction. Confluent HeLa cells grown in 10 cm diameter dishes were treated with TRIzol reagent (Ambion), according to manufacturer's recommendations. Briefly, after cells were harvested by trypsinization and washed twice with PBS, cells were incubated for 5 min in 1 ml TRIzol per dish. 200 µL chloroform per ml TRIzol were then added and the mixture was incubated at room temperature for 2-3 min. After centrifugation at 12,000xg for 15 min at 4°C allowing phase separation, ~600 µL of upper aqueous phase containing RNA was recovered. 500 µL isopropanol per ml TRIzol was added to the

aqueous phase and incubated at room temperature for 10 min to allow RNA precipitation. RNA precipitate was recovered by centrifugation at 12,000xg for 15 min at 4°C, and washed once with 75% ethanol (v/v). After air drying of the pellet, RNA was resuspended in 50 µL RNAse-free water for 15 min at room temperature. RNA concentration was measured with a Nanodrop device and adjusted at 500 ng ml^{-1}.

Reverse transcription. Production of cDNA from RNA was performed with QuantiTect Reverse Transcription Kit (Qiagen, 205311), according to manufacturer's recommendations. Briefly, traces of genomic DNA were first eliminated, followed by reverse transcription for 30 min at 42°C. cDNA was obtained from 1 µg RNA and stored at -20°C.

Primer design. Primers for qPCR were designed using the 'NCBI Primer Designing Tool'. The four transcript variants of CD166/ALCAM (NM_001627, NM_001243280, NM_001243281, NM_001243283) were targeted with the following primers: forward primer, 5'-TTCTGCCTCTTGATC TCCGC-3'; reverse primer, 5'-AGGTACGTCAAGTCGGCAAG-3'. The three transcript variants of AP2M1 (NM_004068, NM_00102520, NM_001311198) were targeted with the following primers: forward primer, 5'-TGCCATTGATGACTGCACCT-3'; reverse primer, 5'-GTGTTCAGTGGGGTTGG GAT-3'. ACTB (single transcript variant: NM_001101) was used as housekeeping gene.

qPCR. qPCRs were performed with QuantiTect SYBR green PCR kit (Qiagen, 204143) according to manufacturer's recommendations, on StepOnePlus (Applied Biosystems) real-time PCR machine. Efficiency of primers was comprised between 90 and 110%.

STxB internalization

Transport of STxB to perinuclear Golgi was performed as previously published[4]. Briefly, cells (treated with specified siRNAs) were seeded in 4- or 24-well plates 16-24 h before the experiment, in order to reach subconfluence the day of the experiment. The day of the experiment, plates were transferred on ice and cells were washed with ice-cold serum-free culture medium. Cy3-labeled STxB (STxB-Cy3; 50 nM) was bound to cell surface on ice in serum-free culture medium for 20 min. After extensive washes with ice-cold serum-free culture medium to remove unbound toxin, cells were placed at 37°C in pre-warmed serum-free culture medium for 45 min to allow STxB endocytosis and transport to Golgi apparatus. Cells were then fixed at 37°C, permeabilized and immunolabeled for Giantin as perinuclear Golgi marker. Samples were imaged by wide field microscopy using tile scanning method to increase the number of cells observed.

STxB tubulation

The observation of plasma membrane STxB-induced tubules was performed as previously published[4]. Cells were seeded in 4-well plates 16-24 h before the experiment, in order to reach subconfluence the day of the experiment. The day of the experiment, cellular ATP was depleted as previously described[5] to inhibit fission mechanisms that process STxB-induced plasma membrane invaginations. In brief, cells were incubated for 15–20 min at 37°C in ATP depletion medium (PBS^{++} supplemented with 10 mM 2-deoxy-D-glucose and 10 mM NaN_3). Plates were then placed on ice and washed with ice-cold ATP depletion medium. STxB-A488 (200 nM), followed by anti-CD166 antibody (5 µg ml^{-1}) were sequentially bound to cell surface on ice in ATP depletion medium for 15 min each. After extensive washes with ice-cold ATP depletion medium, cells were put back to 37°C in pre-warmed ATP depletion medium for 15 min. Cells were then fixed, permeabilized, incubated with secondary antibody, and mounted as previously described (see '*Light microscopy*' paragraph). Samples were imaged by confocal microscopy.

Gold nanoparticle functionalization for Fluid-FM/confocal experiments

Gold beads of 400 nm diameter (Aldrich, 742090) were functionalized with fluorescently labeled His-tagged Gal8 and Gal1 (Gal8-Cy3 and Gal1-Cy5, respectively) following a three-step protocol. Briefly,

gold nanoparticles were incubated overnight with a 90:10 mixture of PEG-OH alkane-thiol (Prochimia, TH001_m11.n3_0.2) and PEG-NTA alkane-thiol (Prochimia, TH007_0.02), followed by 1h incubation with 40 mM Ni_2SO_4. The samples were finally incubated for 1h with either Gal8-Cy3 or Gal1-Cy5 in PBS, resulting in an oriented protein immobilization on the gold surface via His-tag chemistry. Samples were washed by centrifugation and re-suspended in fresh buffer after each functionalization step. The presence of Gal8-Cy3 or Gal-Cy5 on the surface of the gold beads was confirmed by laser scanning confocal microscopy, and only nanoparticles exhibiting a fluorescence signal were selected for the live cell experiments. For control experiments, gold beads functionalized with Alexa Fluor 700-NHS ester dye (Invitrogen) were also prepared, by overnight incubation of the beads with 10 mg ml^{-1} cysteamine (Sigma), followed by direct dye coupling via NHS in $NaHCO_3$ buffer (0.5 M, pH 8.35).

Quantification of cargo uptake from confocal images

Confocal images were quantified using Icy software. Briefly, regions of interest (ROIs) were drawn around each cell, manually or automatically using 'HK-Means' segmentation method (with manual adjustments) when cells were stained with HCS CellMask Deep Red (Invitrogen). Within each cellular ROI, bright spots corresponding to endocytic structures were automatically detected over dark background using the 'Spot Detector' plugin. The plugin performs image denoising by computing wavelet adaptive threshold (WAT) on the union of all ROIs present in an image. This automatic thresholding was then manually adjusted, depending on the size of the spots: for each independent experiment, we chose scale 2 and adjusted sensitivity empirically. In addition, we added a size filter to discard small spots up to 2-pixels. As an output, we obtained the number of endocytic structures within each cell and their respective intensities, that we summed to obtain the total uptake of the cargo per cell. For each experiment, data were normalized to the control condition (set as 100%).

Quantification of STxB transport to Golgi apparatus

Wide field images were quantified using ImageJ/Fiji software. First, DAPI channel was used to count the number of nuclei per image, which indicates the number of cells. To do so, after converting this channel to 8-bit and processing with smooth function, an automatic thresholding was performed with the 'Otsu' built-in method (dark background). Nuclei too close of each other were segmented using the watershed method. Thresholded and segmented nuclei images were submitted to particle analysis, excluding structures smaller than 1.3 μm^2 size or at the edges. At least 500 cells were counted for each condition and for each biological replicate. Next, a mask of Golgi structures was produced by automatic detection of signal in the Giantin-labelled channel in order to produce a mask. Briefly, after converting this channel to 8-bit and processing with smooth function, an automatic thresholding was performed with the 'Moments' built-in method (dark background). The thresholded images were then submitted to particle analysis, excluding structures smaller than 4-pixel size or at the edges. The ROIs of Golgi structures were saved and used as a mask on the corresponding STxB-positive channel images. First, background was substracted from STxB channel using a rolling ball radius of 50 pixels. Then, Golgi ROIs were applied on the STxB channel and the mean gray value of toxin fluorescence in Golgi was extracted for each image. Mean gray values were corrected according to the level of STxB binding to cell surface in each siRNA condition, measured in flow cytometry, and normalized to the control siRNA condition (set as 100 %).

Quantification of co-localization

Quantification of co-localization between two channels was performed using tools previously described[4]. Briefly, an object-based method was used, as implemented in JACoP[6], based on the coincidence between two centroids with a 1- to 2-pixel tolerance. This was achieved in an ImageJ macro by first segmenting the tagged proteins by spot detection in each channel, finding their position, and growing them by dilation to 1- or 2-pixel radius for confocal/TIRF or Airyscan images, respectively.

For confocal and TIRF, images were smoothed (3 x 3 average filter). For classical confocal and Airyscan images, background was substracted (rolling ball radius of 50 pixels). The spot detection consisted in finding maxima in the images using the 'find maxima' plugin of ImageJ, whose noise tolerance parameter was set up visually independently for each channel. The results were expressed as the percentage of co-localizing spots over the total number of spots in one of the two channels.

Quantitative analysis of live cell lattice light-sheet images

Post-processing of raw data volumes was carried out as described previously[7]. Automated detection of clathrin-coated structures or punctate structures of fluorescently labeled cargoes in 3D was performed by numerical fitting with a model of the microscope point spread function (PSF) as described previously[7]. Automated tracking of cargo and clathrin was calculated using the u-track software package[8], included in the cmeAnalysis3D software[7]. We used clathrin and cargo detection positions to map the membrane shape using the Matlab function of alphaShape. Subsequently, we estimated the displacement of each cargo from the membrane position orthogonally inwards of the cell. The distinction between punctate structures diffusing on the membrane and internalized ones whether by clathrin dependent or clathrin independent was made by using the membrane position detection. Meaning, an event was counted as an endocytic event, if an object undergoes a net displacement of at least 150 nm from the initial position inside the membrane proximal zone (Supplementary Figure 2i). The membrane proximal zone was limited by the contour of the alphaShape and a line 400 nm inwards of the cell. To calculate the number of clathrin dependent and clathrin independent events, for every qualified internalization event of the cargo channel, the presence of clathrin was scored using the cmeAnalysis3D software[7]. Only tracks with a duration longer than 8 seconds were used for the analysis. Calculation of lifetime distributions and intensity cohorts were performed as described in details previously[7]. Quantification of LLSM images was performed on raw images. The raw LLSM images, used in Figure 2f,h and in Supplementary Movies 1 and 2, were deconvolved using LLSpy v0.3.7[9] before video rendering in Imaris. LLSpy can be downloaded from https://github.com/tlambert03/LLSpy.

Quantification of spot lifetime at plasma membrane from TIRF images

Time series in TIRF live cell imaging were acquired for total durations of 2 min, and analyzed with Icy software. The analysis was performed using the 'Spot Tracking' plugin and divided in two successive steps: spot detection in 1 or 2 channels, and spot tracking over time. First, bright spots at plasma membrane were automatically detected over dark background using the 'Spot Detector' plugin as above (see '*Quantification of cargo uptake from confocal images*') and saved in the swimming pool. Then, detected spots were linked through time to form tracks using Multiple Hypothesis Tracking (MHT) method, whose parameters were estimated automatically for each time serie. Tracking results were automatically exported to the 'Track Manager' where they could be saved in Excel tables for further statistical analysis and plotting.

Quantification of endoA3 recruitment to plasma membrane

Time series acquired during Fluid-FM/confocal experiments for a total duration of 60 sec were analyzed with ImageJ/Fiji software. For each time series, a circular ROI was defined around the red fluorescent bead (or the apex of the Fluid-FM probe in the case of the bare probe control experiment), and the average fluorescence intensity values from the GFP channel were extracted from each image and finally plotted as a function of time. The intensity versus time plots were then fitted linearly with Origin 2017 v94E (SR1) software, in order to extract the slope of the fitted curve, which was used as comparative parameter to evaluate fluorescence variations among the experimental datasets.

Quantification of gap closure in wound healing assays

Time series acquired during wound healing assays for a total duration of maximum 46 h were analyzed with ImageJ/Fiji software. For each time series, brightness and contrast were manually corrected, followed by treatment with the smooth function (3 x 3 average filter). The gap area (unoccupied by cells) was automatically detected using 'MRI Wound Healing Tool' ImageJ plugin (http://dev.mri.cnrs.fr/projects/imagej-macros/wiki/Wound_Healing_Tool). Values were normalized to the gap area at time 0 and expressed as percentage of gap closure (100% = no unoccupied area left). Average gap closure speed (µm^2 min^{-1}) was extracted from the slope of a linear regression fitted to the plot of gap area in function of time. R-squared were always above 0.95, indicating a good fitting.

Single cell tracking in wound healing images and quantifications

Tracking the migration of individual cells at the edge of the wound was performed manually with ImageJ/Fiji software, using the plugin TrackMate. Cells were followed during 80 frames (corresponding to 800 min), tracks were collected, and several parameters were extracted (migration velocity, xFMI, Euclidian distance).

Quantification of cell-cell adhesion in flipping assays

Wide field images before and after flipping were quantified using ImageJ/Fiji software. After processing images with smooth function, an automatic thresholding was applied (with the default built-in method, over dark background) and adjusted manually. Individual cells too close of each other were segmented using the watershed method. Thresholded and segmented images were submitted to particle analysis, excluding structures smaller than 1-pixel or at the edges. At least 1000 cells were counted before flipping for each condition and for each biological replicate. Cell counts before flipping were considered as a reference for each condition and biological replicate, and cell counts after flipping were expressed as percentage this reference.

Supplementary References

1 Lakshminarayan, R. *et al.* Galectin-3 drives glycosphingolipid-dependent biogenesis of clathrin-independent carriers. *Nat Cell Biol* **16**, 595-606, doi:10.1038/ncb2970 (2014).
2 Blouin, C. M. *et al.* Glycosylation-Dependent IFN-gammaR Partitioning in Lipid and Actin Nanodomains Is Critical for JAK Activation. *Cell* **166**, 920-934, doi:10.1016/j.cell.2016.07.003 (2016).
3 Wisniewski, J. R., Zougman, A., Nagaraj, N. & Mann, M. Universal sample preparation method for proteome analysis. *Nat Methods* **6**, 359-362, doi:10.1038/nmeth.1322 (2009).
4 Renard, H. F. *et al.* Endophilin-A2 functions in membrane scission in clathrin-independent endocytosis. *Nature* **517**, 493-496, doi:10.1038/nature14064 (2015).
5 Romer, W. *et al.* Shiga toxin induces tubular membrane invaginations for its uptake into cells. *Nature* **450**, 670-675, doi:10.1038/nature05996 (2007).
6 Bolte, S. & Cordelieres, F. P. A guided tour into subcellular colocalization analysis in light microscopy. *J Microsc* **224**, 213-232, doi:10.1111/j.1365-2818.2006.01706.x (2006).
7 Aguet, F. *et al.* Membrane dynamics of dividing cells imaged by lattice light-sheet microscopy. *Mol Biol Cell* **27**, 3418-3435, doi:10.1091/mbc.E16-03-0164 (2016).
8 Jaqaman, K. *et al.* Robust single-particle tracking in live-cell time-lapse sequences. *Nat Methods* **5**, 695-702, doi:10.1038/nmeth.1237 (2008).
9 Lambert, T. & Shao, L. LLSpy v0.3.7. *Zenodo* (2017).
