# Supplementary material for: Endophilin-A3 and Galectin-8 control the clathrin-independent endocytosis of CD166
Source: Nat Commun. 2020 Mar 19;11:1457. doi: 10.1038/s41467-020-15303-y (PMC7081352; doi:10.1038/s41467-020-15303-y)
Supplement: Supplementary file 3 — Description of Additional Supplementary Files [file 41467_2020_15303_MOESM3_ESM.pdf]

Description of Additional Supplementary Files

Supplementary Data 1: Data from quantitative proteomic analysis of cell surface proteins in conditions of inhibition of clathrin-mediated endocytosis (CME). HeLa cells were treated for 72h with control or µ2-adaptin siRNAs, and quantitative mass spectrometry analysis (iTraq) was performed on isolated cell surface proteins. 489 proteins (with Unused score ≥ 2) were identified, 378 (77.3%) of which were containing 'membrane' in their GO-annotations. Among these hits, 262 (53.6%) were containing the following exact GO-annotations: 'cell surface', 'plasma membrane', 'intracellular space/matrix', 'integral component of plasma membrane' (hits highlighted in green in the table). The iTraq analysis was performed on 4-plexes, where reporters 114 and 115 were assigned to two technical replicates of negative control siRNA-treated cells, and reporters 116 and 117 were assigned to two technical replicates of µ2-adaptin siRNA-treated cells. In the table, fold-change ratios are calculated relatively to the reporter 114 (negative control condition). Ratios highlighted in red indicate hits showing an increased abundance in the µ2-adaptin-depleted condition compared to the negative control, while in blue, they indicate a decreased abundance. The color intensity depicts the significance of the ratios: no color, not significant; light, $*P < 0.05$; medium, $**P < 0.01$; dark, $***P < 0.001$.

Supplementary Movie 1: 3D movie of anti-CD166 antibody endocytosis imaged by Lattice light-sheet microscopy on live cells expressing CLTA-mRFP, related to Figure 1e-h and Supplementary Figure 2i.
Genome-edited U2OS expressing CLTA-mRFP were incubated for 1 min with 5 µg ml^{-1} anti-CD166-ATTO647N antibody and transferred to the Lattice light-sheet microscope for imaging at 30°C. Full 3D volume of 60 planes per U2OS cell was acquired within 3 sec. Spot color indicates the degree of clathrin co-localization, from blue (clathrin negative) to green (clathrin positive). Tracks are depicted in red.

Supplementary Movie 2: 2D movie of anti-CD166 antibody endocytosis imaged by Lattice light-sheet microscopy on live cells expressing CLTA-mRFP, related to Figure 1e-h and Supplementary Figure 2i.
Same conditions as Movie 1: genome-edited U2OS expressing CLTA-mRFP were imaged after a 1 min incubation with 5 µg ml^{-1} anti-CD166-ATTO647N antibody and transferred to the Lattice light-sheet microscope for imaging at 30°C. Full 3D volume of 60 planes per U2OS cell was acquired within 3 sec. This 2D movie shows a side view of a cell slice perpendicular to the detection objective. Insets: note CD166 endocytic events (red) that are negative (left) or positive (right) for clathrin (green).

Supplementary Movie 3: Uptake of fluorescently labeled anti-CD166 in an endoA3-GFP-positive structure, related to Supplementary Figure 5d.
HeLa cells expressing endoA3-GFP were incubated continuously at 37°C with 5 µg ml^{-1} anti-CD166-A647, and observed by live-cell imaging using a spinning disk microscope at 1.078 sec intervals (exposure time: 200 msec for each channel). Note the tubule that extend inwards from cell periphery. Scale bar, 2 µm.

Supplementary Movie 4: Uptake of fluorescently labeled anti-CD166 in an endoA3-GFP-positive structure, related to Supplementary Figure 5d.
Same conditions as Movie 3: HeLa cells expressing endoA3-GFP were incubated continuously at 37°C with 5 µg ml^{-1} anti-CD166-A647, and observed by live-cell imaging using a spinning disk microscope at 1.191 sec intervals (exposure time: 200 msec for each channel). E, Extracellular medium. N, Nucleus. Scale bar, 2 µm.

Supplementary Movie 5: Fission of an endoA3-GFP-positive tubule.
Same conditions as Movie 3: HeLa cells expressing endoA3-GFP were incubated continuously at 37°C with 5 µg ml^{-1} anti-CD166-A647, and observed by live-cell imaging using a spinning disk microscope at 1.191 sec intervals (exposure time: 200 msec for each channel). E, Extracellular medium. N, Nucleus. Scale bar, 2 µm.

Supplementary Movie 6: TIRFM analysis of endoA3-GFP cells incubated with fluorescently labeled anti-CD166, related to Supplementary Figure 5e-g.
HeLa cells expressing endoA3-GFP were incubated continuously at 37°C with 5 µg ml^{-1} anti-CD166-A555, and observed by live-cell imaging using a TIRF microscope at 1 sec intervals (exposure time: 50 msec for each channel). Scale bar, 5 µm.

Supplementary Movie 7: TIRFM analysis of cells co-expressing endoA3-GFP and µ2-mCherry, related to Supplementary Figure 8f.
HeLa cells co-expressing endoA3-GFP and µ2-mCherry were observed by live-cell imaging using a TIRF microscope at 1 sec intervals (exposure time: 50 msec for each channel). Scale bar, 5 µm.

Supplementary Movie 8: TIRFM analysis of cells co-expressing endoA3-GFP and DNM2-mCherry, related to Supplementary Figure 8e.
HeLa cells co-expressing endoA3-GFP and DNM2-mCherry were observed by live-cell imaging using a TIRF microscope at 1 sec intervals (exposure time: 50 msec for each channel). Scale bar, 5 µm.

Supplementary Movie 9: Fluid-FM – confocal experiment with Gal8-coated gold nanoparticles on endoA3-GFP expressing cells, related to Figure 3e.
HeLa cells expressing endoA3-GFP (green channel) were exposed locally to single Gal8-functionalized nanoparticles (red channel) using a Fluid-FM probe. The recruitment of endoA3-GFP to the site of Gal8 exposure was observed by fast scanning confocal microscopy, recording consecutive images for a total duration of 60 s. Scale bar, 5 µm.

Supplementary Movie 10: Fluid-FM – confocal experiment with Gal1-coated gold nanoparticles on endoA3-GFP expressing cells, related to Supplementary Figure 9i.
HeLa cells expressing endoA3-GFP (green channel) were exposed locally to single Gal1-functionalized nanoparticles (red channel) using a Fluid-FM probe. The movements of endoA3-GFP around the site of Gal1 exposure were monitored by fast scanning confocal microscopy, recording consecutive images for a total duration of 60 s. No significant recruitment of endoA3-GFP was observed. Scale bar, 5 µm.

Supplementary Movie 11: Fluid-FM – confocal experiment with bare probe on endoA3-GFP expressing cells, related to Supplementary Figure 9k.
HeLa cells expressing endoA3-GFP (green channel) were exposed locally to bare Fluid-FM probes, as negative control. The transmitted light channel was used to show the exact positioning of the Fluid-FM probe on the cell. The movements of endoA3-GFP around the Fluid-FM probe were monitored by fast scanning confocal microscopy, recording consecutive images for a total duration of 60 s. No significant recruitment of endoA3-GFP was observed. Scale bar, 5 µm.

Supplementary Movie 12: Fluid-FM – confocal experiment with control fluorescent gold nanoparticles on endoA3-GFP expressing cells, related to Supplementary Figure 9j.
HeLa cells expressing endoA3-GFP (green channel) were exposed locally to single fluorescently labeled gold nanoparticles with no protein attached to it (red channel) using a Fluid-FM probe, as a negative control. The movements of endoA3-GFP around the nanoparticles were monitored by fast scanning confocal microscopy, recording consecutive images for a total duration of 60 s. No significant recruitment of endoA3-GFP was observed. Scale bar, 5 µm.

Supplementary Movie 13: Fluid-FM – confocal experiment with Gal8-coated gold nanoparticles on endoA2-GFP expressing cells, related to Supplementary Figure 9l.
HeLa cells expressing endoA2-GFP (green channel) were exposed locally to single Gal8-functionalized nanoparticles (red channel) using a Fluid-FM probe. The movements of endoA2-GFP around the nanoparticles were monitored by fast scanning confocal microscopy, recording consecutive images for a total duration of 60 s. No significant recruitment of endoA2-GFP was observed. Scale bar, 5 µm.

Supplementary Movie 14: Wound healing assay on U2OS cells, related to Figure 4a-e and Supplementary Figure 10a.
U2OS cells transfected with control siRNAs or siRNAs targeting endoA3 and/or CD166 were followed during 46h for wound healing in bright field microscopy. Images were acquired every 10 min. Quantifications are shown in Figure 4a-e and Supplementary Figure 10a. Scale bar, 100 µm.

Supplementary Movie 15: Wound healing assay on SUM159 wild-type cells, related to Supplementary Figure 10b.
SUM159 cells transfected with control siRNAs or siRNAs targeting endoA3 and/or CD166 were followed during 46h for wound healing in bright field microscopy. Images were acquired every 10 min. Quantifications are shown in Supplementary Figure 10b. Scale bar, 100 µm.

Supplementary Movie 16: Wound healing assay on genome-edited SUM159 AP2-GFP cells, related to Supplementary Figure 10c.
SUM159 AP2-GFP cells transfected with control siRNAs or siRNAs targeting endoA3 and/or CD166 were followed during 46h for wound healing in bright field microscopy. Images were acquired every 10 min. Quantifications are shown in Supplementary Figure 10c. Scale bar, 100 µm.

Supplementary Movie 17: Wound healing assay on LB33-MEL cells, related to Supplementary Figure 10d,e.

LB33-MEL cells, wild-type or stably expressing endoA3-GFP, were followed during 46h for wound healing in bright field microscopy. Images were acquired every 10 min. Quantifications are shown in Supplementary Figure 10d,e. Scale bar, 100 µm.
