# Supplementary material for: Endophilin-A3 and Galectin-8 control the clathrin-independent endocytosis of CD166
Source: Nat Commun. 2020 Mar 19;11:1457. doi: 10.1038/s41467-020-15303-y (PMC7081352; doi:10.1038/s41467-020-15303-y)
Supplement: Supplementary file 22 — Reporting Summary [file 41467_2020_15303_MOESM22_ESM.pdf]

nature research

Corresponding author(s): Henri-François RENARD, Pierre MORSOMME

Last updated by author(s): 27/02/2020

Reporting Summary

Nature Research wishes to improve the reproducibility of the work that we publish. This form provides structure for consistency and transparency in reporting. For further information on Nature Research policies, see Authors & Referees and the Editorial Policy Checklist.

Statistics

For all statistical analyses, confirm that the following items are present in the figure legend, table legend, main text, or Methods section.

n/a	Confirmed	
☐	☒	The exact sample size (*n*) for each experimental group/condition, given as a discrete number and unit of measurement
☐	☒	A statement on whether measurements were taken from distinct samples or whether the same sample was measured repeatedly
☐	☒	The statistical test(s) used AND whether they are one- or two-sided<br>*Only common tests should be described solely by name; describe more complex techniques in the Methods section.*
☐	☒	A description of all covariates tested
☐	☒	A description of any assumptions or corrections, such as tests of normality and adjustment for multiple comparisons
☐	☒	A full description of the statistical parameters including central tendency (e.g. means) or other basic estimates (e.g. regression coefficient) AND variation (e.g. standard deviation) or associated estimates of uncertainty (e.g. confidence intervals)
☐	☒	For null hypothesis testing, the test statistic (e.g. *F*, *t*, *r*) with confidence intervals, effect sizes, degrees of freedom and *P* value noted<br>*Give P values as exact values whenever suitable.*
☒	☐	For Bayesian analysis, information on the choice of priors and Markov chain Monte Carlo settings
☒	☐	For hierarchical and complex designs, identification of the appropriate level for tests and full reporting of outcomes
☒	☐	Estimates of effect sizes (e.g. Cohen's *d*, Pearson's *r*), indicating how they were calculated

Our web collection on statistics for biologists contains articles on many of the points above.

Software and code

Policy information about availability of computer code

Data collection	Zen 2.3 sp1 blue edition software (Zeiss) was used for confocal and epifluorescence microscopy image acquisition.
Data analysis	Data analysis was performed using the following commercial or open-source softwares: ImageJ/Fiji v2.0.0-rc-65/1.51u (NIH) and Icy v1.9.10.0 (Institut Pasteur) for image quantifications; cmeAnalysis3D, Matlab 2018a, Imaris 9.3 and LLSpy v0.3.7 for LLSM image quantifications and vizualisation (cmeAnalysis3D software was published previously by Aguet et al. Mol. Biol. Cell 27(22), 3418-3435, 2016 and can be found as part of the Github repertory of llsmtools in https://github.com/francois-a/llsmtools/. cmeAnalysis3D was implemented in Matlab 2018a. LLSpy v0.3.7 was used to deconvolve LLSM data before video rendering in Imaris. It can be downloaded from https://github.com/tlambert03/LLSpy); 'MRI Wound Healing Tool' ImageJ plugin (http://dev.mri.cnrs.fr/projects/imagej-macros/wiki/Wound_Healing_Tool) for quantification of gap closure in wound healing assays. Object-based quantification of co-localization was performed using an ImageJ macro previously described (for more details, see Supplementary Information, 'Quantification of co-localization'). Quantitative mass spectrometry data (iTraq) were analyzed with ProteinPilot Software 4.5 (AB Sciex). Data were collected in spreadsheets with Excel software (Microsoft). Origin 2017 v94E (SR1) software was used to perform linear regressions on Fluid-FM/confocal data. All statistical analyses were performed using Prism v8.3.0 software (Graphpad Inc).

For manuscripts utilizing custom algorithms or software that are central to the research but not yet described in published literature, software must be made available to editors/reviewers. We strongly encourage code deposition in a community repository (e.g. GitHub). See the Nature Research guidelines for submitting code & software for further information.

Data

Policy information about availability of data

All manuscripts must include a data availability statement. This statement should provide the following information, where applicable:

- Accession codes, unique identifiers, or web links for publicly available datasets
- A list of figures that have associated raw data
- A description of any restrictions on data availability

The authors declare that the main data supporting the findings of this study are available within the article and its Supplementary Information files. The source data underlying each graph in Figures and Supplementary Figures are provided as a Source Data file. The mass spectrometry proteomics data generated during this study, and used for the analysis presented in Supplementary Data 1 and Supplementary Figure 1a, have been deposited to the ProteomeXchange Consortium via the PRIDE partner repository with the dataset identifier PXD017526. The sequences of human endoA proteins used in this study are available in Swissprot database with the accession codes Q99962 [https://www.uniprot.org/uniprot/Q99962] (for endoA1), Q99961 [https://www.uniprot.org/uniprot/Q99961] (for endoA2) and Q99963 [https://www.uniprot.org/uniprot/Q99963] (for endoA3). Extra data are available from the corresponding authors on reasonable request.

Field-specific reporting

Please select the one below that is the best fit for your research. If you are not sure, read the appropriate sections before making your selection.

☒ Life sciences ☐ Behavioural & social sciences ☐ Ecological, evolutionary & environmental sciences

For a reference copy of the document with all sections, see nature.com/documents/nr-reporting-summary-flat.pdf

Life sciences study design

All studies must disclose on these points even when the disclosure is negative.

Sample size	Experimental conditions (number of independent experiments, numbers of cells or fields per experiment,...) were chosen according to standard procedures in cell biology research, as well as according to intrinsic variability of the experiments performed. Statistical significance was tested on the corresponding results.
Data exclusions	No data were excluded from the analyses in this study.
Replication	All replicates are reported in the manuscript.
Randomization	Cell samples in this study were not randomized as it is not suitable in this experimental design.
Blinding	Investigators were not blinded during the experiments. Key experiments such as mass spectrometry were discovery methods and no candidate results were hypothesised a priori. Many quantification methods were semi-automated (immunofluorescence, flow cytometry) and therefore blinding was not required.

Reporting for specific materials, systems and methods

We require information from authors about some types of materials, experimental systems and methods used in many studies. Here, indicate whether each material, system or method listed is relevant to your study. If you are not sure if a list item applies to your research, read the appropriate section before selecting a response.

Materials & experimental systems

n/a	Involved in the study	
☐	☒	Antibodies
☐	☒	Eukaryotic cell lines
☒	☐	Palaeontology
☒	☐	Animals and other organisms
☒	☐	Human research participants
☒	☐	Clinical data

Methods

n/a	Involved in the study	
☒	☐	ChIP-seq
☐	☒	Flow cytometry
☒	☐	MRI-based neuroimaging

Antibodies

Antibodies used	The following antibodies were purchased from the indicated suppliers: mouse monoclonal anti-CD166 clone 3A6 (Bio-Rad, MCA1926, 1:200 for immunofluorescence and flow cytometry on human cell lines); goat polyclonal anti-CD166 (R&D Systems, AF1172, 1:20 for immunofluorescence on mouse cell lines); mouse monoclonal anti-CD166 clone B-6 (Santa Cruz Biotechnology, sc-74558, 1:500 for Western blotting); mouse monoclonal anti-TfR (BD Biosciences, 555534, 1:1,000 for Western blotting); rabbit polyclonal anti-α-adaptin (Santa Cruz Biotechnology, sc-10761, 1:1,000 for Western blotting); rabbit monoclonal anti-EGFR (Cell Signaling Technology, 4267, 1:4,000 for Western blotting); rabbit monoclonal anti-endoA1 (Cell Signaling Technology,

65469, 1:100 for immunofluorescence and 1:1,000 for Western blotting); mouse monoclonal anti-endoA2 (Santa Cruz Biotechnology, sc-365704, 1:50 for immunofluorescence and 1:500 for Western blotting); mouse monoclonal anti-endoA2 coupled to Alexa Fluor 488 (Santa Cruz Biotechnology, sc-365704 AF488, 1:50 for immunofluorescence); rabbit polyclonal anti-endoA3 (Sigma Life Sciences, HPA039381, 1:1,000 for Western blotting); rabbit monoclonal anti-giantin (Institut Curie, recombinant proteins platform, A-R-R#05, 1:50 for immunofluorescence); mouse monoclonal anti-dynamin (BD Biosciences, 610245, 1:4,000 for Western blotting); mouse monoclonal anti-clathrin heavy chain for Western blotting (BD Biosciences, 610500, 1:5,000); mouse monoclonal anti-α-tubulin (Sigma, T5168, 1:5,000 for Western blotting); rabbit polyclonal anti-β1-adrenergic receptor (Abcam, ab3442, 1:1000 for immunofluorescence); rabbit polyclonal anti-Galectin-8 (Biorbyt, orb216142, 1:1,000 for Western blotting); rabbit monoclonal anti-ezrin (R&D Systems, MAB72391, 1:1,000 for Western blotting); mouse monoclonal anti-GST (Invitrogen, MA4-004, 1:1,000 for Western blotting); unconjugated secondary antibodies or conjugated to Alexa Fluor 488, 546 or 647 (Thermo Fisher Scientific); anti-mouse and anti-rabbit secondary antibodies conjugated to horseradish peroxidase (Sigma and Dako, respectively). The mouse monoclonal anti-clathrin heavy chain antibody X22 (used at 1:50 for immunofluorescence) was a gift from E. Smythe (homemade antibody, no catalog number).

Validation

All antibodies were provided by the indicated companies/suppliers as validated antibodies. When necessary, additional validations were performed in our lab (Western blotting, flow cytometry, immunofluorescence), using siRNA-treated cells for the depletion of the targeted protein. Data are reported throughout the manuscript and supplementary information files.

Eukaryotic cell lines

Policy information about cell lines

Cell line source(s)

Cell lines used in this study are: HeLa, MEF, U2OS, BSC-1, HMC3, SUM159, LB33-MEL, MZ2-MEL.43, HeLaM. Cell lines were obtained from ATCC or provided by the various collaborators reported in the Methods section of the manuscript. Genome-edited SUM159 AP2-eGFP and U2OS CLTA-mRFP cell lines were gifts from T. Kirchhausen (Harvard Medical School) and D. Drubin (University of California, Berkeley), respectively. HeLaM cells stably expressing Mito-YFP-FRB and alpha-adaptin-FKBP were provided by M. S. Robinson (Cambridge Institute for Medical Research). LB33-MEL and MZ2-MEL.43 were provided by our colleagues from de Duve Institute at UCLouvain (Prof. Pierre van der Bruggen). HeLa and LB33-MEL cells stably expressing endoA3-GFP were generated for this study.

Authentication

No specific procedure was used to authenticate the cell lines.

Mycoplasma contamination

All cell lines used in this study were negative for mycoplasma contamination. Mycoplasma contamination was tested using a PCR assay (VenorGeM OneStep Mycoplasma Detection Kit from Minerva Biolabs)).

Commonly misidentified lines
(See ICLAC register)

No commonly misidentified cell lines were used in the study.

Flow Cytometry

Plots

Confirm that:

☒ The axis labels state the marker and fluorochrome used (e.g. CD4-FITC).

☒ The axis scales are clearly visible. Include numbers along axes only for bottom left plot of group (a 'group' is an analysis of identical markers).

☐ All plots are contour plots with outliers or pseudocolor plots.

☒ A numerical value for number of cells or percentage (with statistics) is provided.

Methodology

Sample preparation

For cell surface staining (Supplementary Figure 4f,g), uptake assays by loss of surface (Figure 1a,b and Supplementary Figures 2a,b, 3a, 8g) and uptake assays by loss of surface upon increasing endoA3 expression levels (Supplementary Figure 3d), HeLa cells labeled with antibodies or ligands were detached by incubation on ice in PBS containing 4 mM EDTA for 10-15 min.
For the generation of the stable HeLa cell line expressing endoA3-GFP, cells were detached by tryptic digestion and recovered in PBS containing 1% BSA and 1 mM EDTA.

Instrument

For cell surface staining (Supplementary Figure 4f,g), uptake assays by loss of surface (Figure 1a,b and Supplementary Figures 2a,b, 3a, 8g) and uptake assays by loss of surface upon increasing endoA3 expression levels (Supplementary Figure 3d), measurements were made with Guava easyCyte (Merck-Millipore) flow cytometer.
For the generation of the stable HeLa cell line, single cell sorting in 96-well plates was performed with a FACSAria III (BD Biosciences).

Software

Guava easyCyte (Merck-Millipore) flow cytometer was runned by GuavaSoft 2.2.3 software and median fluorescence intensities were collected in Excel files for further analyses.
FACSAria III (BD Biosciences) flow cytometer was runned by BD FACSDiva 8.0.1 software, and GFP mean intensity and index from single cell sorting in 96-well plates were collected in Excel files.

Cell population abundance

A single cell population was always analyzed for surface staining and uptake assays by loss of surface (containing at least 50% of total cells). No tracking of specific subpopulations
For the generation of the stable cell line by single cell sorting, the GFP-positive population represented around 19% of total cells.

Gating strategy

For cell surface staining (Supplementary Figure 4f,g) and uptake assays by loss of surface (Figure 1a,b and Supplementary Figures 2a,b, 3a, 8g), a single gate was chosen on the FSC/SSC plot around the cell population, in order to eliminate counts of cell debris. 10,000 cells were systematically counted in this gate. Unlabelled cells were used as negative control for measurement of background signal.
For uptake assays by loss of surface upon increasing endoA3 expression levels (Supplementary Figure 3d), a first gate was chosen on the FSC/SSC plot around the cell population, in order to eliminate counts of cell debris. 10,000 or 30,000 cells were systematically counted in this gate. A secondary gating was applied to divide the cell population selected in Gate 1 into four categories with differential GFP expression levels: Gate M1 (high level, GFP+++); Gate M2 (medium level, GFP++); Gate M3 (low level, GFP+); Gate M4 (no expression, GFP-).
For single cell sorting of GFP-positive cells (generation of stable HeLa cell line), a single gate was chosen on the FSC/SSC plot around the cell population, in order to eliminate counts of cell debris. A second gate was chosen on the GFP/SSC plot to select GFP-positive cells only. Non-transfected cells were used as a control to determine the limit of GFP-negative cells and position the gate around GFP-positive cells in an optimal manner.

☒ Tick this box to confirm that a figure exemplifying the gating strategy is provided in the Supplementary Information.
